# Personalized glucose forecasting for type 2 diabetes using data assimilation

**DOI:** 10.1371/journal.pcbi.1005232

**Published:** 2017-04-27

**Authors:** David J. Albers, Matthew Levine, Bruce Gluckman, Henry Ginsberg, George Hripcsak, Lena Mamykina

**Affiliations:** 1 Department of Biomedical Informatics, Columbia University, New York, New York, United States of America; 2 Departments of Engineering Sciences and Mechanics, Neurosurgery, and Biomedical Engineering, Pennsylvania State University, University Park, Pennsylvania, United States of America; 3 Department of Medicine, Columbia University, New York, New York, United States of America; Johns Hopkins University, UNITED STATES

## Abstract

Type 2 diabetes leads to premature death and reduced quality of life for 8% of Americans. Nutrition management is critical to maintaining glycemic control, yet it is difficult to achieve due to the high individual differences in glycemic response to nutrition. Anticipating glycemic impact of different meals can be challenging not only for individuals with diabetes, but also for expert diabetes educators. Personalized computational models that can accurately forecast an impact of a given meal on an individual’s blood glucose levels can serve as the engine for a new generation of decision support tools for individuals with diabetes. However, to be useful in practice, these computational engines need to generate accurate forecasts based on limited datasets consistent with typical self-monitoring practices of individuals with type 2 diabetes. This paper uses three forecasting machines: (i) data assimilation, a technique borrowed from atmospheric physics and engineering that uses Bayesian modeling to infuse data with human knowledge represented in a mechanistic model, to generate real-time, personalized, adaptable glucose forecasts; (ii) model averaging of data assimilation output; and (iii) dynamical Gaussian process model regression. The proposed data assimilation machine, the primary focus of the paper, uses a modified dual unscented Kalman filter to estimate states and parameters, personalizing the mechanistic models. Model selection is used to make a personalized model selection for the individual and their measurement characteristics. The data assimilation forecasts are empirically evaluated against actual postprandial glucose measurements captured by individuals with type 2 diabetes, and against predictions generated by experienced diabetes educators after reviewing a set of historical nutritional records and glucose measurements for the same individual. The evaluation suggests that the data assimilation forecasts compare well with specific glucose measurements and match or exceed in accuracy expert forecasts. We conclude by examining ways to present predictions as forecast-derived range quantities and evaluate the comparative advantages of these ranges.

## Author summary

Type 2 diabetes is a devastating disease that requires constant patient self-management of glucose, insulin, nutrition and exercise. Nevertheless, glucose and insulin dynamics are complicated, nonstationary, nonlinear, and individual-dependent, making self-management of diabetes a complex task. To help alleviate some of the difficulty for patients, we develop a method for personalized, real-time, glucose forecasting based on nutrition. Specifically, we create and evaluate the computational machinery based on both Gaussian process models and data assimilation that leverages the physiologic knowledge of two mechanistic models to produce a personalized, nutrition-based glucose forecast for individuals with type 2 diabetes in real time that is robust to sparse data and nonstationary patients. Our computational engine was conceived to be of potential use for diabetes self-management.

## Introduction

One promise of data science is the application of elegant solutions to new and important problems. In this way, personal health care can be seen as a prediction challenge: Identifying the disease the patient has contracted—*estimating the current state*, forecasting the disease progression under different interventions—*estimating the evolution of the state*, and therapy—*optimization of feedback to achieve target state*. The ability to predict outcomes is important in selection of treatment. It is also important for self-management of chronic diseases, as it can help individuals to select the most beneficial self-management strategies and improve their health. The increase in data related to individuals’ behaviors and health biomarkers opens an unprecedented opportunity to use computational and data science methods to predict health outcomes based on basic physiological knowledge and individuals’ historical records. However, to be useful in the real world, such methods need to generate accurate and actionable predictions based on realistic datasets that are consistent with current standards for health monitoring.

Data assimilation (DA) [1], an application of filtering theory [2], pairs mechanistic models with data via Bayesian statistics to create a forecast. It has been applied or deployed successfully in fields including atmospheric physics and meteorology [3, 4], engineering [5], statistics and computer science [6, 7], and, in a more limited way, biomedicine [8]. The power of DA is in its ability to incorporate human knowledge into a forecast; in contrast to the more traditional machine learning approaches, DA with mechanistic models does not require extensive datasets and can be personalized and adapted quickly by estimating mechanistic model parameters. As a result, such models can arrive at an accurate forecast with data that are fewer, more sporadic, or incomplete. The use of DA and control theory in medicine is relatively rare and has been applied in limited situations where data are collected in highly controlled *data-rich* environments. Examples include algorithms in implantable defibrillators and pacemakers to cope with irregular heartbeats [9–13], model construction and fitting for prostate cancer treatment [14], epidemiology [15], and the artificial beta-cell or pancreas project designed to manage insulin and glucose for individuals with type 1 diabetes [8, 16–20].

Here we focus on DA for uses related to type 2 diabetes. Diabetes mellitus is a high-impact disease. In 2012, 8.3% of all Americans and 25% of Americans over the age of 65 had diabetes. It is the 7^th^ leading cause of death in the United States, and costs associated with this disease amount to $176 billion in direct medical costs and $69 billion in reduced productivity in 2012. Type 1 diabetes is an autoimmune disease that destroys pancreatic beta cells and typically renders the body unable to produce insulin. Type 1 accounts for 5% of patients with diabetes. The remaining 95% of diabetic patients have type 2 diabetes [21], a disease with complex causes (cf. Fig. 1 of [22]) where the individual remains capable of producing insulin but has elevated glucose levels.

The main approach to diabetes management is maintaining blood glucose within ranges considered safe. For diabetes patients, such maintenance is done through a combination of nutrition management, physical activity, and medication. Hemoglobin A1c (HbA1c), a physiologic measurement that correlates to a three-month average of blood glucose [23], is a clinical indication commonly used to evaluate diabetes management. Elevated HbA1c values above 8% lead to elevated mortality [24], while individuals with HbA1c in the near normal range of 6–6.5% have improved outcomes [25, 26]. These observations have led to the clinical goal of reducing HbA1c to near normal levels of 6–7% [25, 26] when possible, and maintaining below 8% in nearly all circumstances. From the perspective of self-management, HbA1c normality serves as a long-term goal for patients, but has little practical utility in guiding individuals’ daily choices. Instead, individuals with diabetes self-monitor daily blood glucose readings; higher frequency of self-monitoring of glucose has been associated with better glycemic control and improved clinical outcomes [27–29]. The current recommended frequency of glucose self-monitoring for individuals on intensive insulin regimens is 6–10 times daily; however, no clear guidelines are available for individuals who use basal insulin or oral agents [30].

Nutrition-based glucose management is hard to achieve because it is difficult to understand the impact of foods on glucose levels on a meal-by-meal basis [22, 25, 26, 31]. Glucose-insulin dynamics are high-dimensional, nonlinear, oscillatory, noisy, multi-scale, and dependent on many personal and demographic factors [22]. Studies have demonstrated great variability in individuals’ glycemic response to meals with identical nutritional composition [31]. Anticipating the impact of different meals on one’s blood glucose levels is challenging for both individuals with diabetes and diabetes educators [32]. This personal diversity and forecasting difficulty has led the American Diabetes Association to recommend personalized treatment [33]. There is a clear need for tools and mechanisms that assist individuals with diabetes in understanding and predicting the impact of their nutritional choices on glucose levels and identifying approaches to regulating nutrition to minimize its glycemic impact.

The method requirements to implement personalized forecasting for type 2 diabetes (diabetes will refer to type 2 diabetes unless otherwise noted) glucose management differ from data-rich applications because diabetes data are collected primarily by patients and are sparse, irregularly measured, and noisy. A recent study examining fluctuations in glucose levels for non-diabetics in response to nutrition, physical activity, and sleep established feasibility of utilizing machine learning methods when high density data on glucose levels is available through continuous glucose monitoring [31]. However, because continuous glucose monitoring continues to be out of reach for the vast majority of individuals with diabetes, these data-dense methods are not likely to lead to practical solutions in the near future. Because of this gap, we focus on forecasting glucose in scenarios consistent with common self-monitoring practices of individuals with diabetes. We extend DA to clinical settings that lack tight monitoring and are subject to the constraints of collecting data in real-world clinical situations where the data are complex, irregularly sampled, and biased [34].

Glucose prediction in the context of diabetes has been studied by researchers across many disciplines, and has resulted in a diversity of data-driven approaches for personalized glucose forecasting [35, 36]. These efforts have attempted different tasks (e.g. prediction of hypoglycemia [37–39], glucose control [36, 40–45], and physiologic inference [41, 46–49]), and have taken a variety of approaches to identifying suitable models of a diabetic’s glucose system (e.g. nonlinear Weiner model [40, 41, 50], neural networks [42], probabilistic models [38], and mechanistic systems of ordinary differential equations (ODEs) [48, 49]). Many of the desired prediction tasks have been approached by wrapping the aforementioned models in different predictive algorithms and inference schemes (e.g. stochastic filters [44], PI controllers [45, 51], Gaussian process models [52], fuzzy logic [45], and many other machine learning methods [36, 37, 39, 53]). These studies have paved the way towards personalized interventions for people with diabetes, but most studies fail to show their applicability in settings where data are restricted to those with realistic qualities.

A number of recent studies have begun to focus more on this point of realistic applicability. Zitar and Al-Jabali demonstrated that neural networks could be constructed, based on mechanistic ODEs, to fit a large real-world data set of type 2 diabetics, but lacks sensitivity to nutrition, making the approach less useful for personalized nutritional adjustments [42]. Beverlin et al., on the other hand, demonstrated that glucose predictions can be generated with low error and high correlation by fitting a nonlinear Weiner box model to four patients with type 2 diabetes [40, 41]. However, their model was trained with glucose data sampled every 5 minutes by an implantable sensor, which is neither used nor recommended for the majority of people with type 2 diabetes. Barazandegan et al. proposed the use of online sequential Monte Carlo estimation using a mechanistic model of glucose-insulin dynamics in response to nutrition developed by Vahadi et al., and demonstrated that sequential importance resampling particle filter can be used for accurate individualized forecasting, even in the presence of missing and noisy data [44, 46, 48]. However, this work was based entirely on simulated data, rather than free-living biological data. The research presented here aims to bridge the gap in the literature between proposed methods for personalized glucose forecasting and evaluation of those methods on real-world data. We fit a mechanistic model of type 2 diabetes glucose dynamics ([49]) using a simple data assimilation strategy for sequential estimation (unscented Kalman filter [54]), and demonstrate the efficacy of this approach in 3 individuals with type 2 diabetes and 2 individuals without diabetes who only used a smart phone application to capture pre and post-meal glucose readings and descriptions of their meals.

The goal of the research presented here is to generate personalized, accurate, and actionable predictions of glucose in response to nutrition that can assist individuals with diabetes in making *quantitatively informed* nutritional choices. Our solution uses DA to translate the systems physiology knowledge of glucose-insulin dynamics encoded in mechanistic models into a clinical context to create personalized modeling engines capable of generating nutrition-based, post-meal glucose and HbA1c forecasts in real time. These forecasts would then be integrated into mobile applications such as the one developed by Mamykina *et al.* [32]. To make the information useful for individuals with diabetes, these forecasts must: *(i)* use the minimally invasive data that diabetes patients collect in routine care, up to a maximum of 6–10 measurements a day, *(ii)* personalize to the individual and adapt to changes in behavior and health-state, *(iii)* work in real time, and *(iv)* be accurate enough to produce a forecast that can differentiate glucose values that correspond to between 0.5–2% differences in HbA1c values.

Our solution is constructed and evaluated in eight steps. The data assimilation-based solution is developed and internally evaluated in the inital five steps. Then we show how the DA methodology can potentially be generalized using model averaging in step six, compare the DA against non-personalized versions of itself and with machine learning-based forecasting in step seven, and then demonstrate how the DA output could be translated into a more useful form in step eight. In more detail: *First*, we develop the DA framework within the context of diabetes. *Second*, we show that the DA can generate a personalized forecast using data collected routinely by individuals with diabetes. *Third*, the DA predictions are shown to have similar and sometimes higher correlation with measured postprandial glucose levels than predictions generated by Certified Diabetes Educators who rely on their extensive knowledge and experience, and whose predictions, from the perspective of the patient trying to estimate the glycemic impact of their meal choices, represents the current clinical gold standard prediction. *Fourth*, we show that the DA will not only adapt to the individual, but will also adjust with minimal data when the individual changes behavior quickly. *Fifth*, because there is no single model that will provide the best forecast in all situations, we use statistical model selection [55–57] to pick the best model for each individual given their data capture habits and the employed DA strategies. *Sixth*, armed with the individual model performance results and motivated by achieving the most robust and accurate possible forecast, we show how model averaging techniques can be used to improve model forecast performance. *Seventh*, to demonstrate the impact of tracking states and parameters on a forecast and to contrast the mechanism-based DAs with more traditional machine learning techniques, we generate forecasts using DAs with the state and parameter filters turned off in parallel with a dynamic Gaussian process model. And *eighth*, we take initial steps towards establishing glucose level forecast accuracy benchmarks for individuals with diabetes by examining accuracy of individual forecasts generated using DA over a period of time relevant for self-management.

**Roadmap for the rest of the paper.** This paper is not the least complex and parts of it can be read independently of other parts. To help make reading the paper easier, we are including a descriptive outline of the paper. The *methods section* has five components: (i) DA, the mechanistic models, and basic model selection and evaluation tools, (ii) DA-based model-forecast averaging, (iii) dynamical Gaussian process models, (iv) the study designs and data we use in the paper, and (iv) validation methods for the DA and its forecasts. The *results section* has seven components: (i) DA-based glucose state estimation and convergence, (ii) a comparison between DA-based postprandial glucose forecasts and certified diabetes educator-based postprandial glucose forecasts, (iii) personalization of models though DA-based parameter estimation, (iv) model selection and evaluation, (v) model averaging, (vi) a comparison between the Gaussian process model forecasts and the DA where sub-filters, e.g., state and parameter filters, are turned off, and (vii) a consideration of the potential impact of a clinical intervention. The *discussion section* has two large components: (i) a summary of our results and related discussions, and (ii) a more broad discussion of issues related to applying DA in clinical and biological contexts. Many of the results and discussion sections can be read relatively independently assuming the reader understands the relevant methods introduced in the methods section.

## Materials and methods

### Development of the data assimilation framework with two endocrine models relevant for diabetes

Data assimilation (DA) is a data science machine that unifies models with data to reconstruct the model state and provide forecasts. Crudely, DA is a sophisticated interpolation and forecasting scheme. We focus on the use of mechanistic models that represent human understanding of the system dynamics, although versions also exist for unsupervised models as well. Our objectives here are to reconstruct the state of the endocrine system relevant to glucose-insulin dynamics regulation from the sporadic blood-glucose measurements clinically expected from a patient with diabetes. A detailed explanation of the methods we use can be found in S1 Appendix.

Concretely, the objective of a DA filter is to reconstruct and predict state *x*_*k*_ of a system from noisy, sparse measurements *y*_*k*_, where *k* indexes time. Inherent in this process is assumed uncertainty in how well the state *x*_*k*_ is known. Therefore a distribution of state is assumed and tracked. Because of the combination of the nonlinear, high-dimensional dynamics of the endocrine models and the sparsity of the data, we utilize a variant of an unscented Kalman filter (UKF) or Bayesian sigma point processor [7, 58–61] for DA. Critical in our implementation are the capacities to accommodate sparsely and non-periodically acquired data appropriate for diabetic monitoring and non-stationary system parameters consistent with changes in patient behavior and physiology.

#### State estimation

Kalman filter-based methods utilize a prediction-correction scheme to dynamically track and adjust both the system state and its uncertainty to agree with measurements as they are made: the system model $\dot{x}\left(t\right)=f\left(x,w\right)$ as a function of state *x* and parameter *w* is used to iterate the distribution of system state forward in time to produce a *prediction*; the prediction is then *corrected* to both adjust the prediction and collapse its uncertainty. The objective is to determine the best estimate of state, denoted $\hat{x}\left(t\right)$.

The Unscented Kalman Filter serves the function of smoothing the data in the sense of reconstructing a best, de-noised estimate of all state variables from noisy, incomplete measurements made at times {*t*_*k*_}, and to provide extrapolated estimates of state for all times. For times ahead of the most recent measurement at time *t*_*k*−1_, with best estimate ${\hat{x}}_{k-1}$, these extrapolations $\hat{x}\left(t|{\hat{x}}_{k-1}\right)$ (later referred to as *χ*_*o*_, due to its relationship to the central sigma point as described in the Appendix) provide forecasts of future state:

$$\hat{x}\left(t|{\hat{x}}_{k-1}\right)={\hat{x}}_{k-1}+\int_{t_{k-1}}^{t}f(\hat{x},w)dt$$ (1)

$$=F({\hat{x}}_{k-1},w,t-t_{k-1})$$ (2)

The forecast at the next measurement time ${\hat{x}}_{k|k-1}=F\left({\hat{x}}_{k-1},w,t_{k}-t_{k-1}\right)$ is then corrected to agree with measurements *y*_*k*_ in proportion to the difference between this forecast and measurement,

$${\hat{x}}_{k}={\hat{x}}_{k|k-1}+K_{k}\left(y_{k}-{\hat{y}}_{k|k-1}\right)$$ (3)

where ${\hat{y}}_{k|k-1}$ is the projection of ${\hat{x}}_{k|k-1}$ onto the measurement subspace spanned by *y*. The Kalman gain *K*_*k*_ is derived from a linearization of the dynamics’ flow about ${\hat{x}}_{k|k-1}$, and provides correction of all state variables from the subset of ones actually measured. Once corrected, the new best estimate ${\hat{x}}_{k}$ is used to generate revised future predictions until another measurement is made, while $\hat{x}\left(t|{\hat{x}}_{k-1}\right)$ serves as a smooth extrapolation of $\hat{x}$ for *t*_*k*−1_ < *t* < *t*_*k*_. A full description of the UKF can be found in S1 Appendix.

The *unscented* Kalman filter efficiently accommodates nonlinear models by assuming the state is a multivariate Gaussian distribution around the best estimate ${\hat{x}}_{k}$, whose covariance represents the state uncertainty, and then propagating ${\hat{x}}_{k}$ and a representative finite set of symplex points used to approximate the distribution—denoted as sigma points—forward through the dynamics. The spread of the forward iterates of these sigma points then represent the forecast uncertainty, and their covariance is used to compute the Kalman gain at the next correction step. The appendix contains a detailed explanation of the unscented Kalman filter we use here.

The application of DA to biological and clinically relevant models is not in itself novel. The application of DA to type 2 diabetes using data *constrained* to normal everyday patient collection *is* novel because this application poses particular challenges: the clinical environment is data-poor because data collection is strictly constrained to real-world patient self-measurement patterns, the lack of a first principles model allows for multiple possible best models, and the reality of non-stationary health implies non-stationary model parameters. We address each of these issues below.

#### Model choice, evaluation and selection

There are numerous models of glucose-insulin dynamics of varying complexity [62], and it is neither obvious *a priori* which of these will best fit and predict the relevant dynamics from the limited measurements made, nor whether such optimization may be patient specific. For this study we have limited our analysis to the comparison of two models chosen for their balance between modeled mechanisms and limited complexity: First, we use a relatively simple ultradian model [63] with 6 state variables and 30 parameters. This model is a minimal model developed in a non-pathophysiologic context and represents relatively simple physiologic mechanics. Second, to contrast the ultradian model we use the meal model [49] with 12 state variables and 70 parameters. The meal model includes more digestive mechanics, was developed to include both physiologic and pathophysiologic dynamics, and represents a substantial increase in mechanical modeling complexity over the ultradian model. Each of these models are utilized in the DA framework to reconstruct and forecast the glucose dynamics from patient recorded data. The fidelity of the forecasts is then used to differentiate model performance. The full details and descriptions of the models are included in S2 and S3 Appendices.

#### Parameter selection and estimation

Because of differences between individuals and the non-stationary nature of their behavior and health, we personalize the application of these models by estimating and continually refining model parameters in real-time. Parameters are estimated using a parameter filter that is analogous to the state filter where the states are replaced with parameters.

Due to the practical overfitting constraints imposed by sparse data collection, only a small subset of the parameters—*three parameters per model*—are continually refined. We chose the parameters to estimate using physiologic insight and motivation towards the clinical goal of post-meal glucose forecasting by restricting the estimated parameters to those that strongly influence the mean and variance of glucose. Moreover, we chose parameters that are relatively independent of one another to maximize the effective parameter space and reduce parameter interdependency issues within the parameter filter. Although the measurements are quite sparse, they include measurements just before meals, providing a baseline, and measurements taken at various times after meals, helping to capture variation in glucose dynamics. The result is not only a patient-specific model and forecast, but one that adapts over time with changing individual behavior. It may be possible to devise an optimal measurement pattern but we do not address this topic here. Because the models we use embody established physiological systems and their dynamics, the parameters themselves have direct physiological interpretation. We therefore hypothesize that parameter changes detected over time may be useful indicators of changing health, a topic we address in more detail in the discussion.

#### Data assimilation for states and parameters—Methodological summary

We developed a *modified* dual unscented Kalman filter (UKF) that uses two separate filters to estimate states and parameters. The state filter was modeled after Wan and van der Merwe [61], but augmentation of posterior sigma points was done in proportion to the estimated posterior covariance, rather than with respect to an assumed noise term (Appendix, eq. 28-29). The parameter filter was implemented as defined by Gove and Hollinger [58], where prior parameter covariance is inflated by a “forgetting factor” to allow an additional growth of parameter uncertainty proportional to its covariance, rather than with respect to an assumed noise term (Appendix, eq 46-47). In addition, we enforce a positivity constraint on parameter and state sigma points by setting negative elements to the smallest positive value taken by that element across all sigma points. If no positive values are present, we set the element to zero. Negative (non-physical) parameters and states are inappropriate for the models we use, and can lead to problems with numerical integration, which can subsequently erode the quality of the fit. These ad-hoc adjustments can lead to ill-conditioning of the updated covariance—to overcome this, we generate the next iteration of sigma points with only the real part of the covariance square root. These constraints introduce their own weaknesses, and can lead to underfitting through pre-mature parameter convergence in cases where much of the volume of the covariance square root exists in the complex plane. While this approach is certainly suboptimal, we find it useful for ensuring robustness in an algorithm that must perform reliably in an online clinical setting.

#### Clinical variable estimation

The UKF generates continuous smoothed estimates of states and parameters that can be used to compute estimates of clinically relevant measures, e.g., HbA1c. Clinically, HbA1c is linearly related to the mean of the glucose over the previous three months [23] and summarizes glucose control over that time period. Because HbA1c is an averaged value of blood glucose over a 3-months timeframe, individuals can find it difficult to relate this average to any particular meals or other activities that contributed to it. This problem exists largely due to the sparsity of glucose data collection—individuals don’t collect enough measurements to calculate an accurate average over short time periods. The continuous state UKF estimates allow for short term—order hours to months—moving window mean glucose estimates, providing an opportunity for individuals to understand the source of their HbA1c by understanding *quantitatively* the events that compose their mean glucose over short time periods related to nutrition consumption.

#### Data utilized

The data we use in this paper reflect our goals of proving that a DA can: *(i)* successfully forecast glucose based only on data typically available to individuals with diabetes: carbohydrates in meals and sparse, irregularly sampled glucose measurements, *(ii)* personalize to an individual, *(iii)* adapt when the individual changes, and *(iv)* cope with the *data-poor* constraints of real-life diabetes self-monitoring. Because of these goals, we restricted the data to three individuals with diabetes chosen to capture specific disease and measurement characteristics. Participant 1 measured glucose with a frequency at the upper bound of what is common among individuals with diabetes (1 preprandial and 1–3 postprandial) and self-reported a change in their diet over the course of the study. Participant 1 has well controlled diabetes, with pre- and post-study HbA1c measurements of 6.4% and 5.6%. Participant 2 measured blood glucose in accordance with a typical clinical recommendation for individuals with diabetes (preprandial and 2h postprandial) and did not report any changes in their diet over the course of the study; this participant’s HbA1c at the end of the study was 6.4%. Participant 3 contributed the most sparse and irregular dataset, which included irregular records of meals, days with no records, and glucose captured at random times of day often without reference to meals. This case is included to demonstrate the relative success of the DA forecasts in data-poor situations that imply its particular relevance in real-world practice. Moreover, while participants 1 and 2 represent best-case self-monitoring among people with type 2 diabetes, participant 3’s data presents a snap-shoot of are more average to low level of self-monitoring common among people with type 2 diabetes. Participants 4 and 5 do not have diabetes but were participants in a usability study for a later generation of the mobile application used during the collection of data from participants 1 and 2. We have included participants 4 and 5 because they are physiologically distinct from the participants 1–3 and this difference both increases our data set and provides more evidence of the robustness of the methods we develop in this paper.The details of the data and its collection are included in the methods section.

### Model averaging

The model averaged output quantity, $\hat{g}$, is estimated from a set of models *f*_*i*_, a set of continuous and potentially nonlinear functions that translate the model output into a $\hat{g}$-compatible form. Here *g* is the mean of the posterior distribution or the mean of the sigma points output by the DAs, via an optimized weighted sum:

$$\hat{g}=\sum_{i=1}^{M}w_{i}g\left(f_{i}\right)$$ (4)

where we assume *M* models and positive weights *w*_*i*_ that sum to one, or $\sum_{i=1}^{M}w_{i}=1$. Due to this constraint, *M* − 1 weights *w*_*i*_ are sufficient to determine $\hat{g}$; thus, with two models, we seek the optimal value of *w*_1_. There are many methods for selecting the optimal weights where optimal is defined by the set of weights such that $\hat{g}$ minimizes a model evaluation metric, three of which are introduced in the sections that follow.

#### Linear prediction based model averaging

A clinically relevant and intuitive method for selecting the model averaging weights is to maximize the linear correlation between forecasts and measurements over some time period. Or, assuming measurements *y* and forecasts *x*_*i*_ generated by model *i* on a time period *T* we estimate the linear correlation with:

$$y\left(T\right)=\beta (w_{1}x_{1}\left(T\right)+w_{2}x_{2}\left(T\right))+\epsilon$$ (5)

and we select the weight *w*_1_ that *maximizes* the linear correlation, *β*.

#### Mean square error based model averaging

Possibly the most common method for selecting the model averaging weights is to minimize the mean squared error (MSE) between forecasts and measurements over some time period. Or, assuming *N* measurements *y* and forecasts *x*_*i*_ from model *i* collected over a time period *T* we estimate the MSE with:

$$MSE=\frac{1}{N}\sum_{i=1}^{N}{\left(y\left(t_{i}\right)-\left(w_{1}x_{1}\left(t_{i}\right)+w_{2}x_{2}\left(t_{i}\right)\right)\right)}^{2}$$ (6)

and we select the weight *w*_1_ that *minimizes* the MSE.

#### Information criterion based model averaging

The information criterion ([56]) model evaluation method we use here is based on the Kullback-Leibler divergence ([64]). This method focuses neither on prediction, like linear correlation evaluation, nor error, like MSE evaluation, but rather on the overall closeness between the distributions defined by data and model forecast output. The intuitive interpretation of KL-divergence between two distributions *p* and *q* is the information lost when approximating *p* with *q*. In the context of model averaging, we formulate the KL-divergence as:

$$KL\left(w_{1},w_{2}\right)=\int p\left(w_{1}x_{1}\left(T\right)+w_{2}x_{2}\left(T\right)\right)\log\frac{p(w_{1}x_{1}\left(T\right)+w_{2}x_{2}\left(T\right))}{q(y(T))}$$ (7)

where *p*(*w*_1_ *x*_1_(*T*) + *w*_2_ *x*_2_(*T*)) is the kernel density estimate (KDE) of the model averaged forecasts and *q*(*y*(*T*)) is the KDE of the data. We select the weight *w*_1_ that *minimizes* information loss, or *KL*(*w*_1_, *w*_2_).

### Gaussian process regression model

Some previous studies have used non-mechanism-based machine learning methodology to forecast post meal glucose. We do not use continuous glucose monitor data here and therefore cannot directly compare the DA output with what has been done in other studies, but we can use machine learning methods on our data and compare those forecasts with the DA forecasts to some degree. In the same spirit as was used with the UKF—we use standard methods and do not optimize hyperparameters to simulate what is possible in the high-throughput setting—we use a Gaussian Process Model Regression (GPMR) [65] modified for next step prediction [66]. The theory for these regressions is well-known, so we will not discuss it in detail here. However, it is necessary for us to explain the details of the methodology we use for training, forecasting, and evaluating the GPMR. The regression takes two input variables, pre-meal glucose defined as a glucose measurement within 15 minutes of eating and the carbohydrate content of a meal. The output is the post meal glucose forecast that is independent of time and represents a post-meal glucose value. The GPMR will be trained initially on 50 meals; when new meals are encountered, a post-meal glucose forecast will be made, and then that meal will be incorporated into the training set for the next, yet to be observed, meal. In this way, the training set increases with every meal while the post-meal glucose forecast is always made for a single meal as it would be encountered in real time. The post-meal glucose forecast is compared with the first post-meal glucose measurement on a time interval of 45–180 minutes after the meal. Because of these data constraints, the GPMR cannot be used to forecast pre-meal glucose, glucose at random times, or glucose without a meal and without a pre- and post-meal glucose measurement, and therefore there are fewer meals available to forecast. Because of these practical issues, the GPMR uses a different training set than the UKF schemes. As we have mentioned earlier, there is a lot of space for improving both GPMR and UKF or more broadly DA inference machines, a topic we will address in the discussion.

There are two details worth mentioning related to DAs with mechanism-based models and GPMR. First, both the DAs and the GPMR we use in the paper are Gaussian processes in the formal sense [67], but their primary dynamics are generated by very different processes, one mechanistic with a random component and one has only a random component. Second, the DAs generate continuous forecasts and therefore can forecast all glucose measurements regardless of the data available, making them flexible and robust to missing or partially missing data.

### Study design

In this study, we create the mechanistic model-based glucose forecasting machinery with data assimilation that provides real-time, personalized glucose forecasts that adapt to and track an individual patient’s changing state over time, and we validate it on longitudinal time series from five patients. We demonstrate that the DA forecasts, subject to real world constraints of sparse, noisy, real patient-collected data, personalize, adapt over time, and track quickly relative to postprandial blood glucose readings captured by the participants, such that the DA machinery can be used as the engine in a diabetes self-intervention application. Through the DA we translate physiologic knowledge directly to an individual to help them make disease self-management decisions. The DA we use is designed to track, forecast, and correct states and parameters of nonstationary systems in real time without the luxury of an explicit training set. Instead, the algorithm is adjusted to each patient, using the prior data for training and predicting unseen subsequent data points. Similar methodology was used in the DA model averaging construction. The GPMR construction differs as is demanded by the details of the GPMR computation. It is initialized with a training set of 50 meals when possible and then forecasts post-meal glucose given carbohydrate and pre-meal glucose measurements. For the DA, we use two independent mechanistic-model-based DAs to forecast because different individuals, or individuals in changing states, may require different mechanistic models as forecasting engines. We also develop the model selection criteria for evaluating and selecting the most accurate model, given a prediction task, in the results section.

The five independent datasets chosen represent typical self-monitoring scenarios for individuals with type 2 diabetes from three different diabetes studies. The first three participants have type 2 diabetes while the last two participants do not. In addition, we compared accuracy of the DA predictions with accuracy of predictions generated by certified diabetes educators, whose judgement often represents the gold standard with respect to human-based post-meal glucose prediction and is used to arrive at nutritional therapy recommendations for individuals with diabetes. We compare the DA forecast to that of the diabetes educators to demonstrate that the DA can perform as well as the most skilled humans. This evaluation is important because it compares human and machine accuracy for the prediction task that people with type 2 diabetes must perform every time they consume food. We chose an N of five because our goal is to understand and validate the personalized performance of the DA in depth. In this way we performed five independent experiments and compare their outcomes. We have two layers of inclusion/exclusion requirements, those for this paper and those of the original studies where the data originate; the details of the data collection for the diabetes studies are discussed in paragraphs below and the related citations. For this study we chose five individuals with the following characteristics:

(i) more than two measurements over the course of a meal, (ii) relatively dense measurements in time, (iii) *changing patient state over course of measurement.*, (iv) type 2 diabetes.
(i) two measurements over the course of a meal (before and after), (ii) realistic, sustainable measurements in time, (iii) static patient state over course of measurement, (iv) type 2 diabetes.
(i) relatively random measurement times, (ii) sparse measurements in time, (iii) unknown patient state over course of measurement, (iv) type 2 diabetes.
(i) more than two measurements over the course of a meal, (ii) relatively dense measurements in time, (iii) *changing patient state over course of measurement.*, (iv) non-diabetic.
(i) more than two measurements over the course of a meal, (ii) relatively dense measurements in time, (iii) *changing patient state over course of measurement.*, (iv) non-diabetic.

Their data are shown in more detail in Table 1. We did not exclude data outliers because we want to show the DA performance under realistic, real-time circumstances. Each participant is treated as an independent experimental set-up, and the number of glucose measurements represent the number of experiments per experimental set-up.

**Table 1:** Demographics, data characteristics, and medication information for the five individuals we used to evaluate the DA. Each participant is treated as an independent experimental set-up, and the number of glucose measurements represent the number of experiments per experimental set-up.

Demographic and medication information for five individuals used in our study					
ID	1	2	3	4	5
Disease state	DM2	DM2	DM2	no-DM2	no-DM2
Age	40–50	40–50	40–50	40–50	40–50
Meds	metformin	metformin	insulin	none	none
# of glucose measurements	304	211	24	520	322
# of HbA1c measurements	2	1	0	0	0
# of days measured	27	28	15	90	90

#### Diabetes data collection studies

The three datasets included in the analysis were collected during two separate studies with individuals with diabetes. Participant 3 was a participant in a randomized controlled trial of a mobile application for diabetes self-management, MAHI [68]. Participants 1 and 2 took part in a study that investigated how individuals with diabetes reason about personal data collected with self-monitoring technologies [32]. All three datasets included images of meals taken with smart phones, unstructured textual descriptions of these meals, and blood glucose readings captured at different times of day. During the first study (participant 3), the participants were asked to follow their typical glucose testing schedule; as a result, the first dataset included sparse glucose readings taken 2–3 times per day and often irrespective of meals (e.g. only before or only after). In the second study (participants 1 and 2), the participants were explicitly asked to check their glucose readings before meals, and at least once (2 hours postprandial) or more after meals. Consequently, data sets for participants 1 and 2 include considerably more glucose readings, 7–11 daily. Importantly, while the first study was an intervention study, designed to instigate changes in participants’ behavior over its course, the second was a data collection study without an interventional component or expectations for changes in participants’ behaviors. Yet qualitative interviews with participants of the second study, particularly participant 1 suggested that simple participation in the study alerted them to their nutritional choices and led to some changes in their diet (which was consistent with the change in their reported HbA1c). In contrast, participant 2 did not report any changes in their diet. The demographic information of study participants and statistics on the captured data are included in Table 1. Both studies were approved by the corresponding Institutional Review Boards; all participants signed informed consent form prior to participating. In both studies the participants were reimbursed the cost of testing strips.

#### Diabetes educator prediction data collection study

To compare the predictions of the model with best available human-based glucose predictions, we used predictions of postprandial glucose levels collected during our previous study of reasoning with personally generated data [68]. In that study, two Certified Diabetes Educators were asked to review meals, their textual descriptions, macronutrient breakdown of those meals, and glucose readings before and after those meals extracted from data sets collected by participants one and two. The extracted sets included records made during 5 consecutive days. After that, the diabetes educators were presented with records of meals captured during another 5 days (not included in the previous phase) and glucose levels before these meals, and were asked to predict postprandial glucose levels (at 1h and 2h postprandial for participant one and at 2h postprandial for participant two). We previously reported on these results in comparison with predictions generated by individuals with diabetes. Here we use the diabetes educator forecasts, the best available human-generated predictions, as a point of comparison with the DA glucose forecasts. If the DA forecasts are comparable to those of the best human forecasts, the case can be made that the DA forecasts could be potentially made useful to individuals with type 2 diabetes.

#### Non-diabetes data collection study

Data for non-diabetic individuals was collected as part of the pilot study of a smart phone application for forecasting individual’s glycemic reaction to prospective meals based on their previous history. The two individuals whose data was used in this paper were recruited as part of a convenience sample, were both in their early 40s, had no known health issues, varied in gender (one male, one female), and had college degrees. They used the app for 4-6 weeks each to capture all their meals and blood glucose levels before and after each meal (2 hours postprandial) for the duration of the study.

All of the studies were approved by the corresponding Institutional Review Boards; all participants signed informed consent form prior to participating. In all studies that involved collecting blood glucose readings, the participants were reimbursed the cost of testing strips.

### Evaluation, validation, generalizability and usability of the model-based UKF construction

In this paper we address the validation or evaluation, generalizability, and usability of the mechanistic model-based UKF forecasts. The UKF is a prediction/correction machine, meaning that it uses the most recent data to make predictions until the new data point is encountered, at which point the prediction is evaluated and the UKF states and parameters are corrected. This implies a different validation and generalizability situation from what is often encountered in biomedical studies.

*Validation* of the UKF, because of the prediction/correction construction, requires each person to be treated as an independent experimental set-up. Therefore, sample size for validation is the number of meals per person. For validation purposes, we conducted five experiments on different experimental set-ups where the experiments range from having sample sizes of 24 to 520. We validate the model/UKF combination using mean squared error, linear correlation, and a KL-divergence-based distance on a per-person/experiment basis. Moreover, we compare expert nutritionist forecasts to model forecasts, but this is not so much a validation of the UKF as it is a quantitative validation of the challenges that individuals with type 2 diabetes face in the course of their self management and a demonstration of a way the UKF-based forecast could potentially help individuals with type 2 diabers. The nutritionist is the gold standard baseline for *human-based* prediction of the glycemic impact of a chosen meal. Therefore, when we compare the nutritionist forecasts with the model-based forecasts we are comparing machine-forecasting to human-based forecasting to demonstrate the potential usefulness of the UKF framework. It is important to understand that the nutritionist is not the gold standard by which we evaluate the UKF, the glucose measurements serve that purpose.

*Generalizability* moves beyond validation of the UKF and addresses the likelihood, for a population, that the UKF equipped with one or another of the mechanistic models would be applicable. Relative to generalizability to the population of human beings, we have a sample size of five including three individuals with type 2 diabetes and two individuals without type 2 diabetes. While these five individuals are rather diverse and have different disease states with only five people we cannot claim that the models or the UKF will generalize to all people. However, this is not the purpose of this paper; before justifying imposing the UKF on a larger population, it is important to validate and understand, on an individual level, the accuracy of the UKF forecast and how well the UKF can personalize to different individuals.

*Usability* moves beyond validation but in a different direction than generalizability. Usability addresses the usefulness, to a person with type 2 diabetes, of the UKF output. To address usability one must address the consistency of the accuracy of the UKF output and the nature of the presentation of the UKF output. For example, the usability is often quantified based on the impact of the presentation of the forecast on the patient state as the study progresses. The retrospective nature of this paper and the limited population size prevent us from addressing usability in earnest here. What we do instead is investigate the building blocks for a potential diabetes management intervention. We do this to help put the UKF forecasts in a more clinical context and justify that the UKF forecasts can be of potential use for a diabetes intervention. But a first set in any usability study is to understand the accuracy of the information given to the users—this paper was written with this step in mind.

## Results

### Glucose state estimation using data assimilation

**Fig 1:**
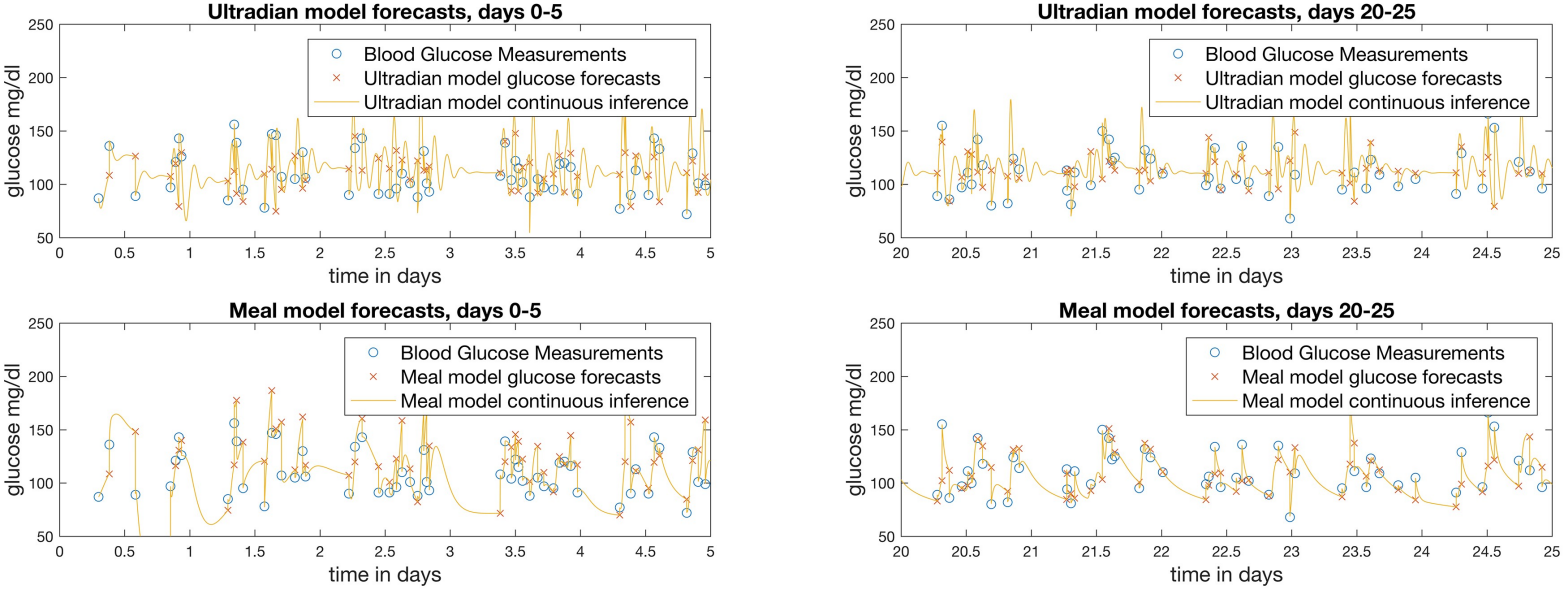
Glucose measurements, forecasts, corrections, and continuous forecasts of both models for days 1–5 (left) and 20–25 (right) for participant 1. These representations of the tracking and forecasting processes performed by the UKF provide an intuitive understanding of the estimation procedure, as well as for the dynamics of each model. Forecast errors are generally larger for both models during early stages of training (days 1–5), but while both models produce similarly reasonable forecasts of each discrete measurement (which we establish quantitatively), both models predict qualitatively *different* off-data continuous forecasts, represented by the lines, over the same time periods and subject to the same measurements; this difference demonstrates that the models represent different physiology.

The visualization of the personalized glucose forecasts from both models, the continuous glucose imputation, and the convergence to better forecasts as more data are accumulated for participant 1 is shown in Fig 1. To demonstrate the model forecasts at the beginning and how they improve over time, we have included plots of forecasts for days 1–5 and 20–25. In these plots, the DA receives data as if it were collected in real time, makes forecasts, corrects the glucose trajectory and model parameters according to each individual measurement, and then repeats this process. There are four features in Fig 1 of note. *First*, the models seem to converge over time and provide better forecasts. *Second*, the models appear to generate accurate forecasts. *Third*, the glucose dynamics of both model-based DA outputs lie within the range of measured glucose values. And *fourth*, the off-data model dynamics differ substantially between models despite the similar accuracy of both model forecasts.

**Fig 2:**
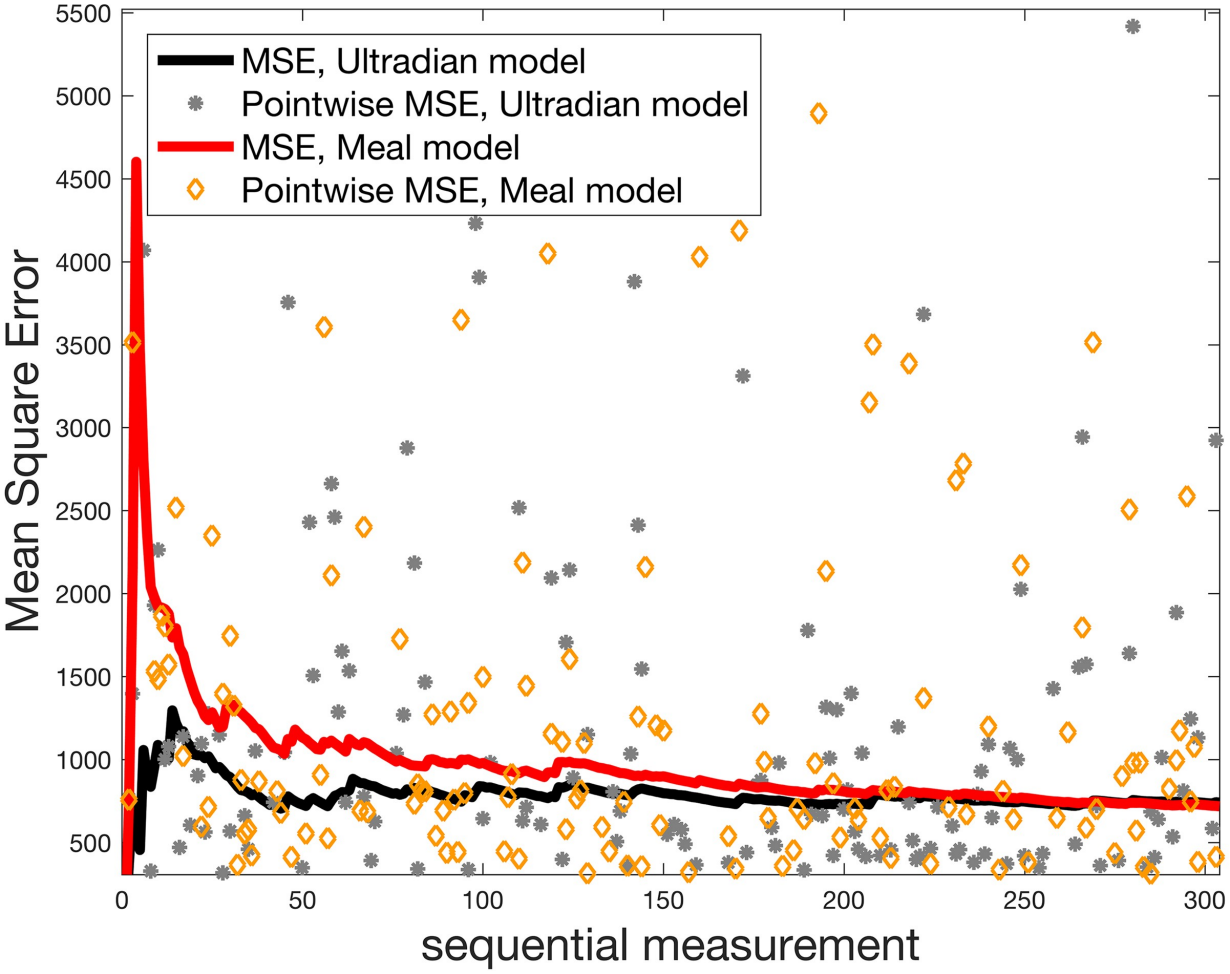
Mean squared error *integrated* over time after day 7 for participant 1 for both ultradian and meal models. Notice: both models show convergence in mean squared error over time; after 200 measurements the integrated mean squared error for both models does not differentiate them; and as the number of measurements increase, the majority of the errors are below the integrated mean squared error value.

While the DA output looks reasonable in Fig 1, it is important to quantify the goodness of fit. Fig 2 shows the mean squared error between the *real-time* DA forecasts and the measurements over time for both models. There are three notable features in Fig 2 *First*, the models converge after about 50 measurements—at 8 glucose measurements per day, convergence is achieved with about one week’s worth of data. *Second*, while the ultradian model does better initially relative to the mean squared error, the models provide approximately equally accurate forecasts given two to three weeks of data. And *third*, the mean squared error improves over time—most of the point-wise mass of the mean squared error lies below the mean, implying that much of the post-convergence error is generated by a few large-error forecasts.

### Comparing DA glucose forecasting to Certified Diabetes Educator forecasts

We have established that the DAs converge and generate accurate forecasts; the next question is to compare predictions generated by the model with those generated by human experts, here Certified Diabetes Educators. To be clear, we are not using the diabetes educators as a gold standard for the DA but rather as a gold standard for human-based forecasting of the glycemic impact of individual meals. There are two reasons why we want to compare diabetes educators to the UKF forecasts. First, while human-based glycemic forecast evaluation is not the topic of this paper, it is important that we demonstrate the difficulty of the task we are asking of patients, every time they eat, by showing how well a highly educated/trained individual performs at the task of forecasting glycemic impact of a given food intake. Second, we need to have a proof in principle from a clinical standpoint that the DA predictions of by-meal glycemic impact were comparable or better than to what a trained diabetes counselor would forecast for individual meals; i.e., demonstrating that the DA can do as well as the best trained humans at forecasting glycemic impact. By doing this we can establish a proof in principle, from the clinical standpoint, that with little tuning the DA could automatically match or beat the state of the art in clinical care.

Before we present the results, there are some important caveats related to this analysis and comparison. *First*, comparison we present here is relatively underpowered. We compare only 14 experiments—meals—for three experimental set-ups, participant one’s glucose forecasts and measurements one hour and two hours post-meal and participant two’s glucose forecasts and measurements two hours post meal. Recall that the dataset for participant 1 typically had two postprandial blood glucose measurements, while the dataset for participant 2 typically had only one postprandial blood glucose measurement. Because of this, we do not have one-hour correlations for participant 2. Participant 3 did not have enough data to calculate statistically significant one or two-hour post meal correlations between measured and forecasted glucose. Some of the quantities we would like to calculate are not resolvable with these data, but we can make enough good estimates to make useful and important comparisons. *Second*, the educators generally estimate the glycemic impact of a meal not post-meal glucose at a precise time, making pin-point forecasts an unusual task for them. Because of this, we might expect the linear correlation—the quantity that captures accuracy the estimate of the mean—to be well forecast by the diabetes educators while the MSE—the quantity that captures point-wise forecast error—to be more difficult for the diabetes educators. *Third*, the DAs are continuous models and generate a glucose forecast every minute regardless of the data present. The continuous glucose dynamics are oscillatory—like that of an oscillator driven by nutrition and damped by insulin and metabolism—so the model output can differ by 100 mg/dl within a 15 minute interval over the first two hours after food has been consumed. The oscillations driven by meal consumption for both models can be seen in Figs. 1 in S2 and S3 Appendices respectively. Because of this, we might expect that the model forecasts would have high MSE and low linear correlation with measurements at one hour post-meal and low MSE and high linear correlation at two hours post-meal.

Table 2 has the results of the comparison between the diabetes educator post-meal glucose forecast accuracy and the DA post-meal glucose forecast accuracy. The *primary conclusion* is that the DA forecasts compare favorably to the forecasts of the certified diabetes educators given the same data. This conclusion can be reached though five observations drawn from Table 2. *First*, the DAs do not have good point-wise accuracy one hour after a meal as expected. *Second* the DAs have better point-wise accuracy two hours after the meal in comparison with the point-wise accuracy of the diabetes educators, as expected. *Third*, the diabetes educators have the same point-wise accuracy at one and two hours after the meal, but their point-wise accuracy is higher than we had expected. *Fourth* it is difficult to compare the linear correlation—the estimate of the post-meal mean—between the post-meal measurement and forecasts of the diabetes educators and the DAs for participant one because of data sparsity. And *fifth*, the linear correlations between the between the two hour post-meal measurement and forecasts of the diabetes educators and the DAs for for participant two were roughly comparable.

**Table 2:** A comparison between the post-meal glucose forecasts of Certified Diabetes Educators and the DAs for a subset of the meals consumed by participants one and two. The model forecasts compare well with expert forecasts. There is no one-hour correlation for participant 2 because participant 2 did not have one-hour post-meal glucose measurements.

Comparing DA forecasts with certified diabetes educator forecasts			
Patient	Forecast	MSE	LC
P1, 1 Hr, 14 Meals	Ultradian Model	2400	-0.44, p = 0.11
	Meal Model	1900	0.13, p = 0.65
	Expert 1	870	0.36, p = 0.2
	Expert 2	660	0.62, p = 0.02
P1, 2 Hr, 14 Meals	Ultradian Model	520	0.18, p = 0.54
	Meal Model	290	0.73, p = 0.003
	Expert 1	890	0.14, p = 0.64
	Expert 2	900	0.14, p = 0.55
P2, 2 Hr, 14 Meals	Ultradian Model	570	0.77, p = 6e-4
	Meal Model	1000	0.58, p = 0.02
	Expert 1	470	0.72, p = 0.003
	Expert 2	340	0.75, p = 0.001

It is, of course, relatively easy for us to improve the DA performance such that the DAs will outperform the diabetes educators if we tune the DA hyper-parameters by hand to a given individual. But, one of the key points of this paper is to demonstrate that the DAs, with no human intervention, can *automatically* generate a glucose forecast that is of similar or better quality as a highly trained human expert. Given the likelihood that knowledge of Certified Diabetes Educators greatly exceeds knowledge of a typical individual with diabetes, it is likely that DA-generated predictions will be more accurate than those generated by individuals with diabetes and therefore of potential help to individuals with type 2 diabetes.

### DA-based personalized medicine: Model personalization and adaptation

People, their metabolism, their behavior, and their presentation of diabetes are both *personal and dynamic*. To be useful, the DA must both personalize to the individual and adapt to changes in the individual over time. The models forecast glucose based on three features: glucose measurements, carbohydrate measurements, and model state defined by the parameter settings. While glucose and carbohydrate dynamics change on fast time scales (order minutes to hours), the baseline endocrine state and the model parameters that represent it are slower moving variables with dynamics evolving on the order of days to months.

We estimate three parameters for each model every time a new measurement is encountered. The ultradian model parameters we personalize include the exchange rate for insulin between remote and plasma compartments, *E*, the volume of insulin distribution in the plasma, *V*_1_, and the time constant for plasma insulin degradation *t*_1_, all of which affect the glucose mean and variance [69]. The meal model parameters that we estimate include the insulin volume, *V*_*I*_, and the glucose kinetics parameters *k*_1_ and *k*_2_, both of which are related to the severity of diabetes and also affect the glucose mean and variance.

**Fig 3:**
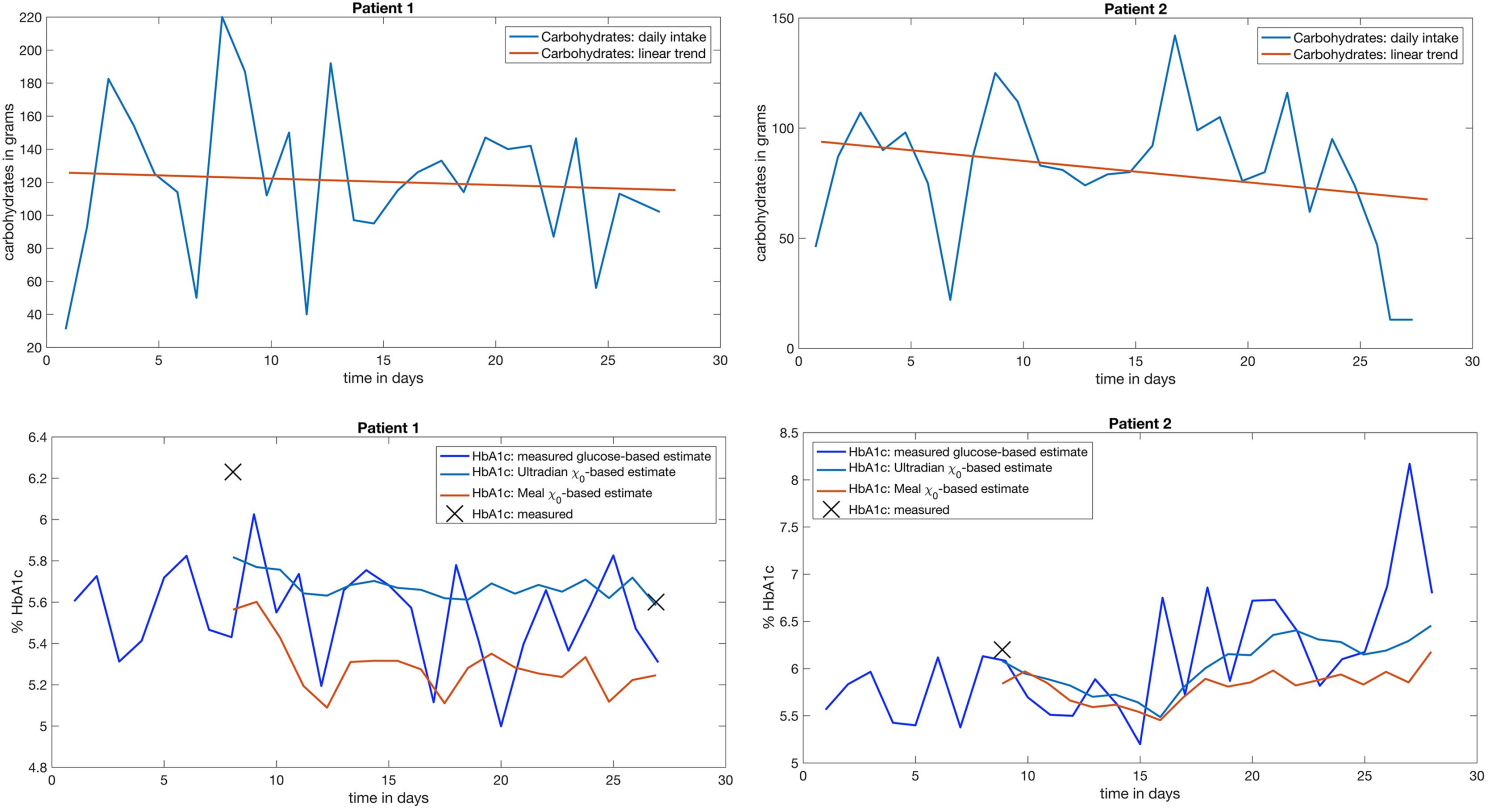
**Top row**—carbohydrate consumption in grams averaged every day for participants 1 and 2; participant 1 reported making dietary changes during the course of the study, with a particular focus on reducing the amount of consumed carbohydrates, while participant 2 did not report any changes in their diet during the study. **Bottom row**—HbA1c measurements for participants 1 and 2, measured-glucose-based HbA1c estimate, and the associated continuous DA generated *χ*_*o*_-based *daily* HbA1c smoothed estimates for both the meal and ultradian models; the *χ*_*o*_-based HbA1c forecasts are accurate *and track broad glucose trends* in all cases and *the ultradian model HbA1c forecast for participant 1 predicted both the trend and the final measured HbA1c value*. The initial underestimate of participant 1’s HbA1c is due to a combination of two factors: (i) the initial HbA1c measurement is taken at a time where the model error is high because it is early in the sequential estimation process and (ii) the model training set does not include much data prior to the first HbA1c measurement.

To initially visualize and evaluate the adaptation of the models, we will first focus on HbA1c as a long-term clinically-motivated proxy for glucose. The relationship between HbA1c and glucose is understood physiologically and is well documented empirically [23]—HbA1c is linearly related to the mean glucose value averaged over the previous 90 days. The time period necessary to estimate HbA1c can be considerably shorter than 90 days, and the lower bound on the length of this time window is unknown. The problem of predicting HbA1c can then be framed as the challenge of determining the true mean glucose over such a time window. However, because we are focusing on opportunities for *real time* decision support, it is of greater interest to relate short term behaviors to long-term outcomes. As such, we explore methods for computing accurate daily glucose averages that can be easily converted to proportional HbA1c values. Concretely, these daily HbA1c estimates represent the expected HbA1c outcome for a patient if they were to maintain that day’s mean glucose over 90 days. In Fig 3, we evaluate daily HbA1c estimates by computing daily mean glucose levels for patient-recorded measurements and continuous off-data model forecasts.

Fig 3 shows the carbohydrate consumption of participants 1 and 2, daily HbA1c estimates, and real HbA1c measurements. Embedded in Fig 3 are five important results. *First*, by observing the changes in carbohydrates, we can verify that participant 1 consistently reduced their carbohydrate intake over the course of the study. Participant 2 also reduced their carbohydrate consumption, but not consistently, and by the end of the study their mean glucose was trending upwards. Participant 1’s HbA1c changed in the course of the study, potentially due to the change in carbohydrate consumption. For participant 2, the single HbA1c collected during the study is consistent with the HbA1c estimates from both glucose measurements and forecasts. *Second*, the results make evident the challenges related to estimating HbA1c on a time scale of days from only glucose measurements *taken in the course of self-management*. The culprit is sparsity of measurements; estimating a reliable and low-variance mean given 3–8 numbers taken from an oscillatory signal is non-trivial. Importantly, mean glucose may be more stable across days than is made apparent to patients by infrequent measurements.

*Third*, the *smoothed*, fast time scale HbA1c estimate computed from the continuous glucose forecasts provided by the DA is more reliable, stable, and accurate than an estimate based on glucose measurements collected with frequencies *consistent with common practices of individuals with diabetes*. It is possible that continuous or carefully selected glucose readings could be as good or better than DA smoothing, however, neither of these approaches are feasible for the majority of individuals with T2D. For participant 1, DA is able to overcome the data-quality deficit: the ultradian model forecasts participant 1’s second HbA1c measurement nearly perfectly. The models track mean glucose well and are able to provide *real-time forecasts* that give us the ability to compute real-time smoothed mean glucose and HbA1c estimates, thus providing both a short-term glucose forecast and a long-term forecast of meals’ impact on glycemic control. However, which of these model outputs are most useful for self-management remains an open question. *Fourth*, both models track the HbA1c with expected accuracy. The glucose-measurement estimated HbA1c is well tracked by a smoothed DA-based HbA1c estimate. *Fifth*, the DA-based HbA1c forecasts seem to have a roughly 5-8 day lag behind nutrition as can best be observed in the plot of participant 2’s HbA1c and carbohydrate dynamics. The carbohydrate and HbA1c trends are roughly 5 days out of phase. This suggests that, given the constraints imposed by real-world frequency of data collection, the DAs change the basic endocrine state on the order of 5–8 days, or 50 measurements. This rate of adaptation is consistent with the initial convergence rate that required roughly 5–8 days or 50 measurements to converge. However, the key point is that the DAs continuously adapt and correct the endocrine basic state, improving forecasting accuracy, on the order of 50 measurements or 5–8 days, a speed that is likely fast enough to be useful in the context of diabetes self-management.

**Fig 4:**
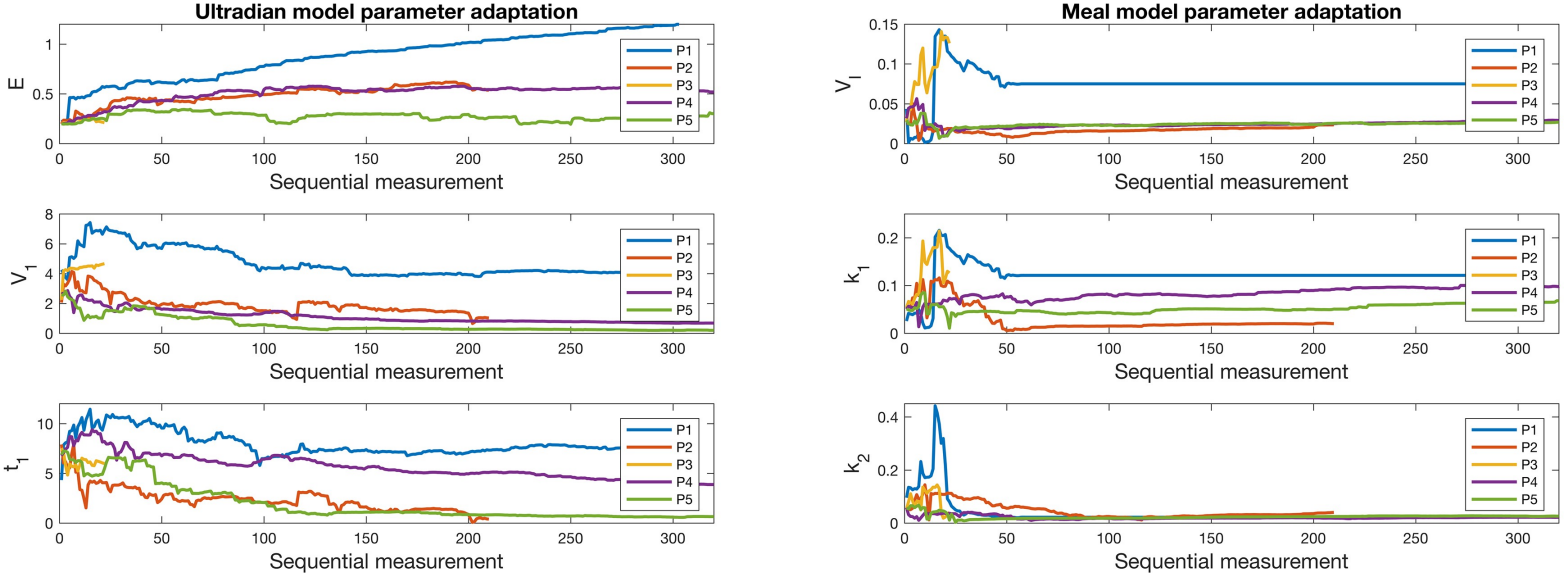
Convergence in time, i.e., *personalization* of the DA, for three parameters of ultradian model (left) and three *different* parameters for the meal model over the course of measurements for all five patients. Recall that the models have few overlapping parameters so it is not possible to compare parameters between models. Notice: (i) each model converges to a different state, depending on the patients, personalizing to the individual, (ii) visually, initial convergence of the parameters requires about 50 data points, (iii) patient 1, who continuously changed their behavior, had parameters that evolved in time in contrast to patient 2 whose behavior did not evolve and whose parameters did not change appreciably after 50 data points, and (iv) when using the meal model with patient 1, the potential for underfitting due to the sigma-point constraint in the parameter filter can be observed at measurement 50—recall that the sigma-point constraints were added to ensure robustness of the UKF, and that there exist methods for rectifying such issues, which we do not address.

The convergence and adaptation of the model parameters estimated by the DA are shown in Fig 4. This plot shows the evolution of three estimated parameters as new measurements are encountered. There are four notable results in these figures. *First*, the model parameters generally converge to a curve with much slower or no change after approximately 50 measurements—this is consistent with the convergence rates we observed in the mean squared error in Fig 2. *Second*, both models adapt continuously to participant 1 who *consistently decreased* carbohydrate intake. The models show the adaptation to this change primarily in a single parameter—the exchange rate for insulin between remote and plasma compartments, *E*, of the ultradian model and the estimated insulin volume, *V*_*I*_, of the meal model. *Third*, participant 3 did not have enough measurements to converge to a personalized state, but it is clear that the convergence was underway because several of the parameters appear to be leveling off to a constant state, following a *qualitatively similar* path as participant 2’s parameter convergence. This helps establish a measurement constraint, participant 3 has only 24 measurements and while the DA can issue better forecasts than a linear regression, the DA appears to need closer to 50 measurements to personalize. And *fourth*, the participants were all in relatively different health states and the models evolved through distinctly different health states, providing evidence of personalization.

### Model selection: Identifying the best model to drive the DA for a person and circumstance

We have no first principles type understanding of systems physiology, instead mechanistic models are constructed using empirical observation and stylized facts as is often done in economics [70]. Our object of study here, the endocrine system, is a complex, high dimensional physiologic system that depends on spatial scales from the molecular to the societal and time scales from microseconds to decades. We do not know which model with what components will most accurately represent a given person in a given circumstance. Because of this, to achieve accurate personalized forecast of glucose, it is important to include several different models and to develop a methodology for selecting the most useful model for a given set of circumstances, or employ a model-averaging scheme [55–57]. For simplicity, here we have restricted our choices to two mechanistic models and focus on model selection, rather than model averaging.

There is neither an objectively best model nor an objectively best model evaluation methodology [55]. To present a holistic picture and triangulate an understanding of what the different models do well and how they differ, we use four model selection techniques. Our *first* model evaluation—mean glucose—is likely the most intuitive, but not the most robust or accurate evaluation metric. *Second*, as previously discussed and seen in Fig 2, we use the pointwise mean squared error. *Third*, we use an information criterion technique—the Kullback-Leibler (KL)-divergence between the kernel density estimate of the real measurements and the kernel density estimate of the forecasts [64]. The *fourth* method we use to evaluate model performance is the linear correlation between DA forecasts and measured glucose values.

**Fig 5:**
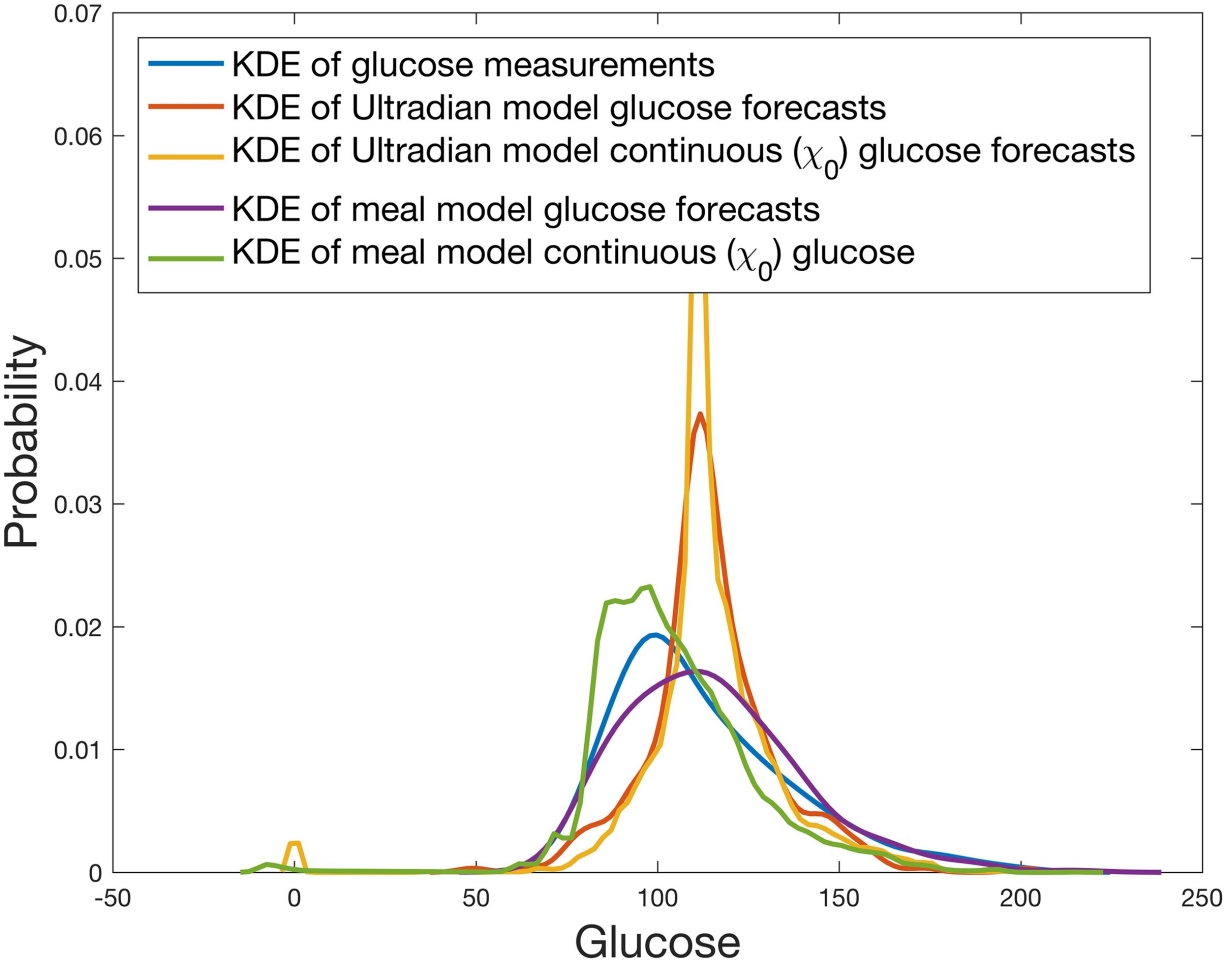
The kernel density estimates of the probability densities (PDFs) of glucose measurements and a variety of forecasts after day 7 for participant 1 for both ultradian and meal models. These PDFs are used to estimate the KL-divergence between the kernel density estimate of measured and model forecasted glucose values. For this patient the PDF generated by the meal model appears to more closely resemble the PDF generated by the measured glucose than the PDF generated by the ultradian model. Importantly, the PDFs of the ultradian and meal models are distinct and different from one another, implying differing physiologic processes and mechanisms present.

Forecast means in Table 3 show measured and DA-based mean glucose estimates for all five participants. While all models produce reasonable results, the ultradian glucose forecasts generate the most accurate mean glucose estimates. This result is surprising because the ultradian model is the simpler model and does not reproduce the measured glucose distribution as accurately as the meal model, as seen in Fig 5. The accuracy of the continuous off-data forecast of the ultradian model is important because the continuous forecasts are produced every few minutes, generating enough glucose values to render an accurate *short term*, e.g., 24 hour HbA1c forecast that can illustrate long-term impact of immediate nutritional choices, an option that is usually unavailable.

**Table 3:** Model evaluations for individual DA estimates is presented. KL designates the KL divergence between the kernel density estimate of measured and DA-output glucose, LC designates linear correlation, potentially with a lag. Notice: *(i)* all models produce reasonable results, the ultradian mean glucose forecasts are the most accurate, *(ii)* the accuracy of the continuous forecast is important because the forecasts are produced every minute, generating enough glucose values to render an accurate *short term* HbA1c forecast that patients can immediately relate to their nutrition intake, *(iii)* the metrics do not always select the same best model, *(iv)* no single model is the representative of all patients, and *(v)* both models are capable of representing and forecasting patients.

Model evaluation metrics of the DA forecasts					
Participant	1	2	3	4	5
Disease state	T2D	T2D	T2D	no-T2D	no-T2D
Mean glucose, measured	113 ± 25, (*σ*)	127 ± 32, (*σ*)	119 ± 16, (*σ*)	92 ± 17, (*σ*)	101 ± 16, (*σ*)
Mean glucose, ultradian model forecast	**115** ± **15**, (*σ*)	124 ± 12, (*σ*)	115 ± 9, (*σ*)	90 ± 13, (*σ*)	**101** ± **12** (*σ*)
Mean glucose, ultradian model *continuous* forecast	116 ± 14, (*σ*)	**129** ± **16**, (*σ*)	**120** ± **17**, (*σ*)	**91** ± **13**, (*σ*)	100 ± 10, (*σ*)
Mean glucose, meal model forecast	105 ± 28, (*σ*)	120 ± 26, (*σ*)	126 ± 37, (*σ*)	87 ± 22, (*σ*)	97 ± 27, (*σ*)
Mean glucose, meal model *continuous* forecast	101 ± 15, (*σ*)	118 ± 28, (*σ*)	132 ± 35, (*σ*)	76 ± 19, (*σ*)	86 ± 17, (*σ*)
KL, Ultradian model and glucose	**0.54**	0.29	3.9	0.65	2.6
KL, Meal model and glucose	1.2	**0.11**	**1.4**	**0.18**	**0.20**
MSE, Ultradian model and glucose	**680**	**950**	**250**	**260**	**280**
MSE, Meal model and glucose	730	1300	2800	540	590
LC(1HR) Glucose with itself	0.3, *p* = 0.07	—	—	0.1 *p* = 0.4	0.12 *p* = 0.3
LC (2HR) Glucose with itself	−0.02, *p* = 0.9	0.07, *p* = 0.6	—	−0.13 *p* = 0.11	0.18 *p* = 0.13
LC Ultradian model forecast with glucose	0.12, *p* = 0.04	**0.33**, *p* = 6.0*e*^−7^	0.10, *p* = 0.67	**0.38**, *p* = 9.0*e*^−20^	0.32 *p* = 5.0*e*^−9^
LC Meal model forecast with glucose	**0.24**, *p* = 3.5*e*^−5^	0.31, *p* = 6.0*e*^−6^	**-0.47**, *p* = 0.03	0.36 *p* = 3.0*e*^−17^	**0.38**, *p* = 4.0*e*^−12^
LC(1HR) Ultradian model forecast with glucose	**0.31**, *p* = 0.03	—	—	**0.39**, *p* = 0.001	**0.32**, *p* = 0.006
LC(1HR) Meal model forecast with glucose	0.20, *p* = 0.16	—	—	0.28 *p* = 0.02	0.29 *p* = 0.01
LC(2HR) Ultradian model forecast with glucose	−0.11, *p* = 0.39	0.43, *p* = 0.004	—	0.44 *p* = 3.0*e*^−9^	0.18 *p* = 0.13
LC(2HR) Meal model forecast with glucose	0.07, *p* = 0.55	**0.55**, *p* = 8.6*e*^−5^	—	**0.46**, *p* = 7.3*e*^−10^	**0.46**, *p* = 4.0*e*^−5^

The KL-divergence is a measure of the distance between two probability density functions—here the difference between glucose probability density function estimates. Small KL-divergence implies the *graphs* of the two probability density functions are similar, indicating good model estimates. Visual intuition of the KL-divergence can be gleaned from Fig 5 that shows the kernel density estimates of the glucose measurements and forecasts for participant 1. Formally, the KL divergence is defined by:

$$KL\left(p,q\right)=\int p\log\frac{p}{q}d\mu$$ (8)

where *p* and *q* are probability densities and *μ* is the Lebesgue measure. The KL-divergence between *p* and *q* is interpreted as the information lost when *p* is approximated by *q*. For us, the interpretation becomes more complex because of measurement biases—biases caused by the missing values and the noise associated with them due to the self-monitoring data collection process for an individual with diabetes. Here we compare the kernel density estimates of real, sparse measurements, DA forecasts *restricted to times where there are real glucose measurements*, and continuous DA-based imputation of glucose. The differences between the kernel density estimates for these quantities shown in Fig 5 may be nontrivial. Without continuous blood glucose monitoring, the true continuous distribution of glucose is unknown. Nevertheless, combining the information in Table 3 with what we observe in Fig 5 suggests that while the measurements and model outputs all generate a similar mean glucose value, the *graphs of the kernel density estimates are qualitatively different*. Moreover, it does appear that the kernel density estimate of the meal model based forecasts most closely resembles the kernel density estimate of the measured glucose values, while the mean of the ultradian model forecasts is closer to the mean of measured glucose. This highlights why there is not an optimal method for model selection. Contrasting different evaluation metrics, different integrals, allows for a clearer picture of how well the models are performing.

The full summary of the model evaluation shown in Table 3 presents a consistent vision. For participants 1, 2, 4 and 5 the ultradian model generally matches or outperforms the meal model, except when it comes to the linear correlation between forecast and measurement likely because the ultradian model represents an estimate of the mean. For participant 3, the ultradian model outperforms the meal model. This implies that even in our simplified situation, there is clearly no best model for all patients and model evaluation metrics, although the ultradian model does appear to be the better choice unless estimation of a distributional or linear correlation based quantity is the desired goal. Most importantly, both models perform quite well, implying that any choice have the potential to yield accurate forecasts.

### Model averaging—Blending models to achieve a more accurate forecast

#### Finding an optimal model average relative to different quality metrics

**Fig 6:**
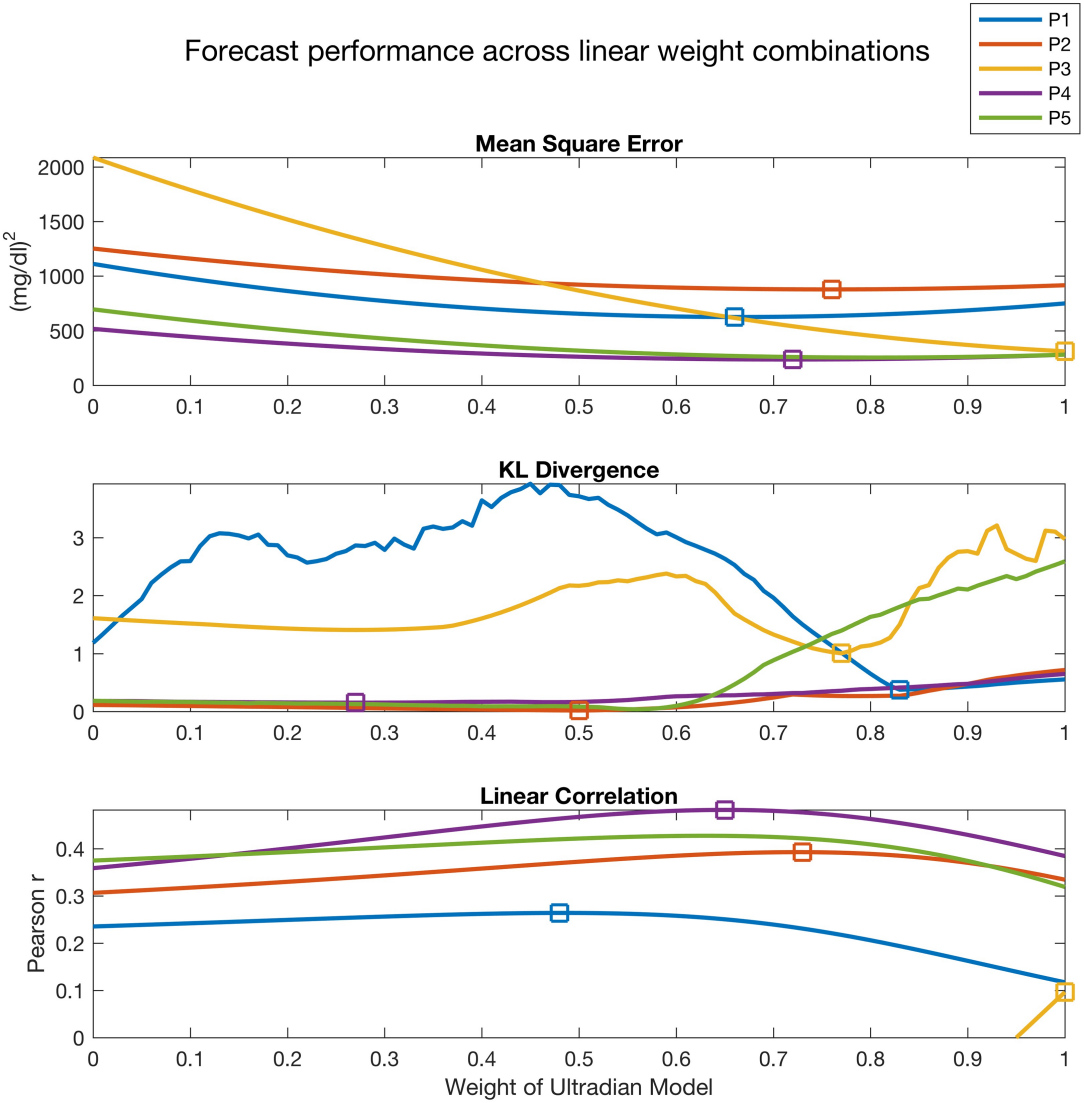
Mean square error, KL-divergence, and linear correlation for the entire set of forecasts for each participant was plotted with respect to ultradian model weight. Boxes represent the points at which each metric is optimized. Note that most optima exist between 0.5–0.8. Both mean square error and linear correlation have single inflection points, whereas KL-divergence takes on many local minima. The presence of optimal weights that are far from both 0 and 1 indicate the potential value of model averaging in this context.

The first question to ask is what value of *w*_1_, when applied uniformly in retrospect to all forecasts, optimizes model evaluation metrics. Fig 6 depicts the relationship between the averaging parameter, *w*_1_, for ultradian model and meal model output versus mean square error, KL divergence, and linear correlation. All metrics for all patients are optimized (shown by the squares in the plot) by weighting a portion of each model. The optimal weights for MSE and LC are both between 0.5–0.8, favoring a larger proportion of the ultradian model. The KL divergence also shows *local* minima in this range for all patients, although KL also tends to be low for *w*_1_ = 0 (i.e. meal model forecasts only). These results push three conclusions. First, model averaging, even in relatively simple implementations, can improve forecast accuracy. Second, model averaging re-enforces what we observed in Table 3, that choosing an optimal model can depend on the quality metric being optimized. And third, for clinical applications, it will be necessary to carefully choose the quality metric that will most usefully help patients and clinicians make bedside decisions.

#### Model averaging by optimizing a single quality metric

A straight-forward method of picking model weights for a forecasting task is to use the weighting that optimizes a particular metric for all previous forecasts. The efficacy of this approach can be evaluated by looking for improvements not only in the optimized metric, but in other metrics as well. In Table 4, we report the MSE, LC, and KL evaluations of forecasts that were generated with model weights that were selected at each forecasting step to optimize one of the three metrics for all past data.

**Table 4:** Model averaging with sequential weight selection, based on mean square error, KL-divergence, and linear correlation, is evaluated for all three performance metrics and compared to the performance of individual models. Values are bolded to designate the model that performs best under each criterion for each participant. While MSE-based averaging consistently outperformed the individual models, the ideal scheme varies across patients.

Evaluation of DA-based model forecast averaging				
Patient	DA/averaging	MSE	LC	KL
P1	Ultradian Model	750	0.12	0.55
	Meal Model	1110	**0.24**	1.2
	MSE-based **average**	**640**	0.21	**0.25**
	LC-based **average**	850	0.23	2.6
	KL-based **average**	810	0.18	3.2
P2	Ultradian Model	920	0.33	0.72
	Meal Model	1250	0.31	0.11
	MSE-based **average**	**905**	**0.37**	0.75
	LC-based **average**	990	0.36	0.18
	KL-based **average**	960	**0.37**	**0.07**
P3	Ultradian Model	310	0.1	3.0
	Meal Model	2100	-0.47	**1.6**
	MSE-based **average**	480	-0.25	3.3
	LC-based **average**	**300**	**0.13**	3.1
	KL-based **average**	370	0.0088	3.2
P4	Ultradian Model	280.6004	0.3846	0.6477
	Meal Model	520	0.36	**0.18**
	MSE-based **average**	**240**	**0.48**	0.29
	LC-based **average**	270	0.46	0.23
	KL-based **average**	360	0.39	0.49
P5	Ultradian Model	280	0.32	2.6
	Meal Model	700	0.3751	**0.18**
	MSE-based **average**	**260**	0.38	3.0
	LC-based **average**	330	0.35	0.95
	KL-based **average**	280	**0.44**	0.91

In all cases except P3, MSE-based averaging outperformed both models in MSE, and the LC-based averaging forecasts outperformed both models in LC for two of the five patients. *KL-based model averaging underperformed noticeably*, since it produced an inferior KL-divergence when compared with the meal model alone for four of five patients. Moreover, KL-based model average forecasts tended to have higher MSE as compared with other methods.

The results in Table 4 demonstrate that *the ideal model averaging scheme varies across patients*. Forecasts for P1 were best optimized by an MSE-based average or MSE and KL, whereas forecasts for P3 were best optimized for MSE and LC by the KL-based average. Optimal averaging schemes for P2, P4, and P5 varied more greatly depending on the metrics we wish to optimize. Overall, the MSE-based average seems to be a good choice for everyone except P3, who collected far fewer data points than the other four participants. KL-based averaging achieved high ranking results for P2 and P5, but, interestingly, other methods do a better job of improving the KL divergence.

#### Time variation in model averaging weights

**Fig 7:**
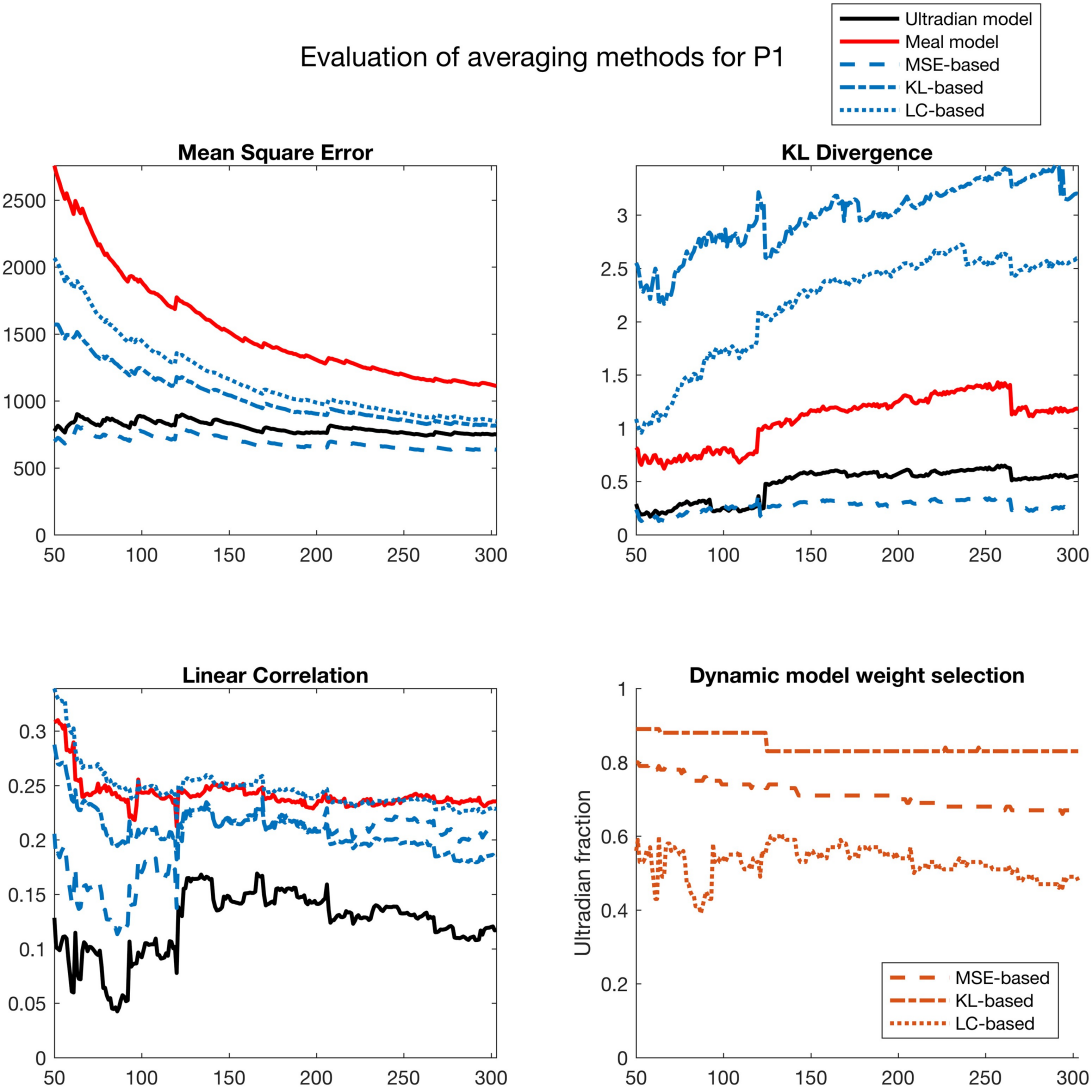
The progression of model averaging performance is shown for P1, along with the weights selected by each method at each forecasting step. MSE-based and LC-based averaging show immediate improvements in their respective quantities, and are able to strike a balance between the high linear correlation of the meal model and the low MSE of the ultradian model. For P1, only the MSE-based average improved long-term KL divergence. In addition, the MSE-based average achieved better error than the ultradian model while nearly doubling the correlation of its forecasts with real measurements. This pattern becomes evident within the first 75 forecasts, demonstrating the feasibility and utility of doing model selection across model averaging modalities.

It is also informative to explore how each of these evaluation-based weighting schemes would function in an applied setting by plotting their efficacy in time, along with the weights selected at each point by each method. This allows us to evaluate *if and when an ideal model averaging metric is knowable*. In Fig 7, we see that for P1, it was beneficial overall to favor ultradian forecasts. When optimizing MSE, the ultradian forecasts proved more valuable, whereas optimizing LC required a more even pairing across models. The performance of model averaging in time for P1 shows that better model averaging methods revealed themselves within 50–100 measurements (MSE-based and LC-based averaging already achieved improved MSE and LC scores after 50 measurements compared to the individual model performance). After 100 measurements, all participants’ forecasts were improved by model averaging methods that, indeed, remained valuable throughout the rest of the patient timeline. In some cases, an additional 50 forecasts were needed to perform an informed model selection across averaging approaches, but most often, the first 100 measurements were sufficient to determine the optimal model selection strategy.

### The impacts of mechanisms and filtering analysis

We have shown that the DA converges given our clinical data constraints, the DA personalizes to an individual, and that averaging models can help produce a better forecast. To demonstrate the impact and necessity of the state and parameter filters and the mechanistic UKF set up as well as comparing the DA to standard non-mechanism-based forecasting machines, we must turn off the various filters and implement different forecasting machinery and observe how the forecasts accuracy changes. Table 5 shows the mean forecasted glucose and the mean square error and KL-divergence between the measured and the forecast glucose values. This table prompts two comparisons—the DA with different filters included and the dual UKF DA with the GPMR.

**Table 5:** Comparing filters and a non-mechanistic forecasting machine. The different filter configurations include all filters off, state-only filters and dual (state and parameter) filters enabled. The non-mechanistic forecast is provided by the Gaussian Process Model Regression (GPMR). In all but one case, the dual UKF based on the ultradian model provides the most accurate forecast—although model averaging in Table 4 provides an even more accurate forecast. While the GPMR does have a potentially useful MSE, it is not as useful for tracking the mean glucose state because it is trained only on glucose measurements surrounding meal times.

Model and filtering comparison						
Patient	Model	Filter	Mean BG	Forecast mean BG	MSE	KL
P1	Ultradian	no-filter	115 ± 15	109 ± 15	770	0.04
		state filter		108 ± 18	780	0.29
		dual filter		115 ± 15	680	0.54
	Meal	no-filter		109 ± 15	3600	0.06
		state filter		108 ± 18	2100	0.19
		dual filter		105 ± 28	730	1.2
	GPMR			130 ± 9	630	0.30
P2	Ultradian	no-filter	127 ± 32	107 ± 6	1800	0.14
		state filter		107 ± 8	1800	0.35
		dual filter		124 ± 12	950	0.29
	Meal	no-filter		107 ± 6	1800	0.07
		state filter		107 ± 9	1700	0.03
		dual filter		120 ± 26	1300	0.11
	GPMR			140 ± 17	1200	0.08
P3	Ultradian	no-filter	119 ± 16	107 ± 7	330	0.36
		state filter		107 ± 7	330	1.33
		dual filter		115 ± 9	250	3.9
	Meal	no-filter		107 ± 7	3500	2.9
		state filter		107 ± 7	3500	2.7
		dual filter		126 ± 37	2800	1.4
	GPM			—	—	—
P4	Ultradian	no-filter	92 ± 17	108 ± 17	640	0.60
		state filter		108 ± 17	630	0.97
		dual filter		90 ± 13	260	0.65
	Meal	no-filter		108 ± 17	7000	0.34
		state filter		107 ± 7	5300	2.1
		dual filter		87 ± 22	540	1.4
	GPM			93 ± 4	330	0.57
P5	Ultradian	no-filter	101 ± 16	110 ± 14	500	0.2
		state filter		110 ± 14	500	0.27
		dual filter		101 ± 12	280	2.6
	Meal	no-filter		110 ± 14	4000	0.16
		state filter		110 ± 14	2800	0.14
		dual filter		97 ± 27	590	0.20
	GPM			110 ± 5	350	0.17

To demonstrate the impacts of the state and parameter filters, we consider three DAs: (i) no-filter—we set the initial glucose and nutrition to be the latest measurement and run it forward to the next measurement; (ii) state-filter—were we run the UKF state filter but not the parameter filter, and (iii) the dual UKF state and parameter filters. In this setting the mean for the state and no-filter is mostly determined by the initial parameter settings because the parameters are not changed. There are four results of note. First, the effects of the different filtering constructions was consistent between models. Second, we did not observe much difference between the state filter and the no-filter DAs. Third, the dual filter, which includes parameter correction, provides a substantial improvement over the no-filter and the state-only filters with respect to the accuracy of the mean and pointwise mean square error. Fourth, the KL-divergence is usually minimized by the no-filter and the state filter, but none of the KL-divergences are particularly large.

Comparing the dual UKF powered by mechanistic models with a non-mechanistic machine is difficult to make completely equitable or unbiased, largely because the natural input and training sets for the two different models are so different. The UKF really track and re-aligns the mechanistic model using whatever data are currently available, substituting model data for any missing data maintaining a continuous-time model of the system where as the GPMR is a regression that takes a full input vector and maps it to an output vector. It is possible that with a continuous glucose monitor data such a comparison between the GPMR and the UKF would be more equitable, but this is not the situation we address in this paper. The dual UKF forecasts were generated as previously discussed. The GPMR forecasts were generated using data for meals restricted to the situation where there is a pre-meal glucose taken within 15 minutes of eating the meal, a post-meal glucose taken within 45–180 minutes of the beginning of the meal—the most recent post-meal glucose is always used as the post-meal glucose—and of course carbohydrate estimates. The GPRM is trained on the first 50 meals and when it encounters a new meal, it makes a forecast based on the pre-meal glucose and carbohydrate estimates, and then that meal is added to the training set. The GPMR can be very sensitive to nonstationarity—participants 1 and 2 in particular—and when it is, the its accuracy is highly sensitive to the training window length. We chose the training and forecasting procedure to minimize the dependence on the training window size. Relative to this construction we have four key results. First, the GPMR generally overestimates the mean glucose by a substantial amount, expect for participant 4. Second, the GPMR has a competitive MSE—is is usually the second lowest compared with the dual UKF using the ultradian model. The KL divergence for the GPMR is low and usually preforms about as well as the no-filter DA, likely because both the processes are Gaussian without too much imposed mechanistic structure. And fourth, if we instead use a fixed window size, unless the meal training window is short—less than 50 meals—the GPMR performs considerably more poorly than any of the dual UKF implementations. We will save more analysis of the GPMR for another time.

### Building blocks for a diabetes management intervention

The underlying expectation of this research is that generating accurate predictions of individuals’ glycemic response to particular meals can inform their decision-making and improve their glycemic control. Our final objective, then, is to examine different ways of communicating predictions generated with DA that provide actionable information and convey different degrees of confidence and precision. The natural output of the model is a continuous forecast with a potentially indefinite endpoint, and there exist many different ways to present this information to individuals with diabetes in order to inform their decisions. Previous research suggested that visualizing these projections in a graphical way, as a continuous curve, can lead to improved understanding [71]. Another possibility is to condense this continuous forecast into a projected range that communicates to the individual the anticipated glycemic impact of the meal. While traditional static error terms communicate average forecast uncertainty, they do not convey higher-order information (e.g. extreme values, velocity) that are relevant to understanding the case-specific effects of nutrition. Instead, we consider different ways of generating interval predictions that reflect expected fluctuations in blood glucose and have the added benefit of communicating forecast uncertainty. Specifically, we examine three dynamic forecast intervals derived from continuous DA output and characterize them by comparing the magnitude of their bounds with an empirical estimate of the confidence interval they represent.

**Fig 8:**
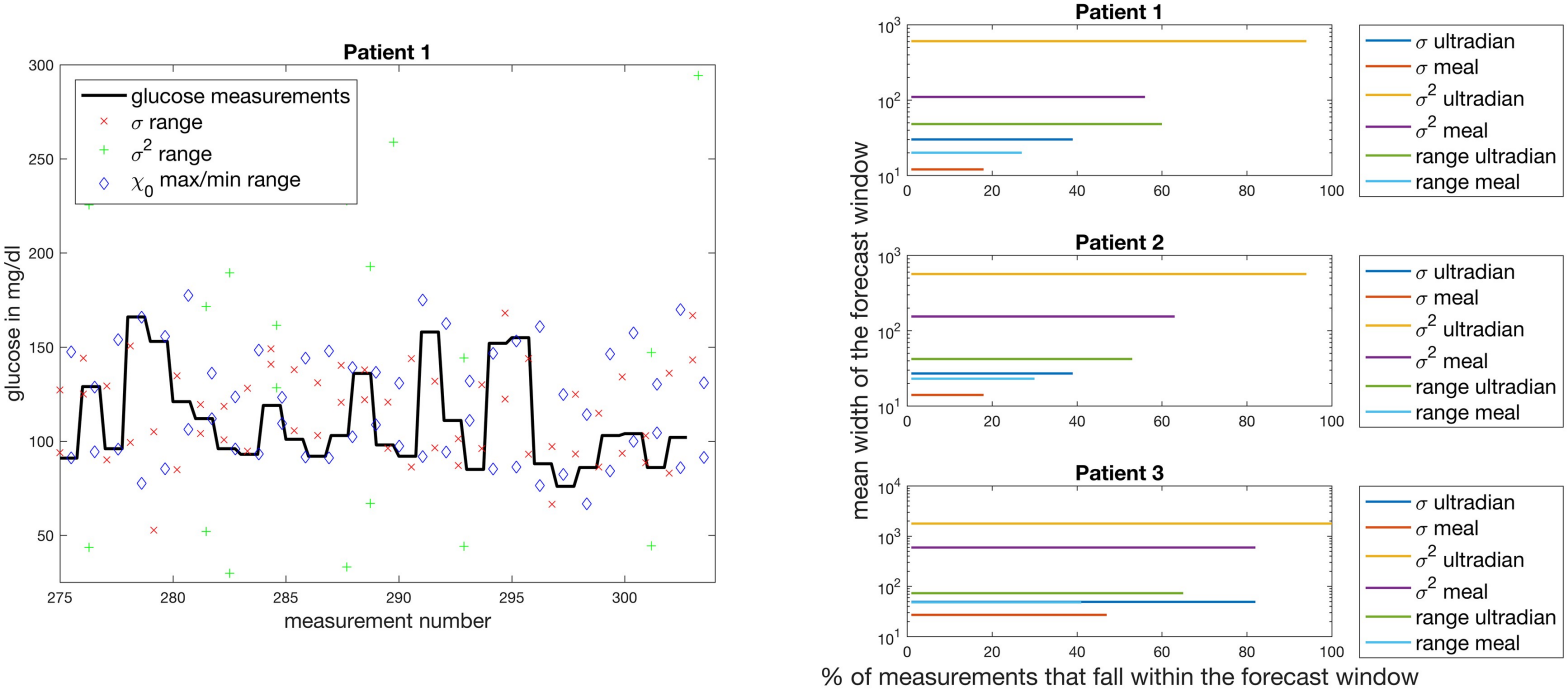
Practical forecast accuracy of DA forecasts can be captured by calculating the percentage of measurements that are contained within glucose forecast-derived forecast ranges. Here we consider three such ranges, standard deviation, variance, and range of the glucose forecast 30–120 minutes after a given meal. The *left plot* shows the last 25 glucose measurements of participant 1 and a moving window of the ultradian model-based off-data forecast ranges. From this we can see how the windowed forecast ranges predict the glucose value: the variance is too wide to be useful but contains all measurements, and the range encapsulates proportionally more measurements as the *more narrow standard deviation*. The *right plot* shows the percentage of post meal measurements captures versus with width of the boundary window.

The left plot of Fig 8 shows the last 25 glucose measurements and three model-derived *dynamic* bounds *on a moving window*, ± standard deviation, ± variance, and range (maximum minus minimum) of the continuous ultradian model-based glucose forecast 30–120 minutes after a meal. The right plot shows percentage of measurements captured by those boundaries. Recalling Fig 1, the ultradian model has high amplitude oscillations while the meal model has limited oscillations; this dynamic difference has a profound impact on which model may provide the most useful forecast *boundary*. Generally, the ultradian model forecasts capture a higher percentage of future measurements. The variance is too wide to be useful for any of our participants. The ultradian model range provides a better balance of frequency of accuracy; the dynamic range of ±15–20 capturing 50–60% of future measurements compared to standard deviation of ±15 capturing 40% of future measurements. Here participant 3 is the exception with a standard deviation of ±25 capturing 82% of future measurements. This difference demonstrates the complexity of translating a forecast that is a density into a quantity such as a single number—the ideal range may have to be chosen automatically for a given person. These results are a proof in principle that a plausibly useful forecast range could be created.

## Discussion

The broad goal of this research is to develop new ways to forecast postprandial blood glucose levels given an individual’s preprandial blood glucose level and nutritional composition of a meal, particularly amount of carbohydrates. To achieve this goal we developed a DA and integrated it with two mechanistic endocrine models, a model averaging methodology, and a dynamical Gaussian process model regression, largely for comparison purposes. We can observe the DA *personalizing* the models by estimating model parameters for individuals in real-time with as few as 50 glucose measurements. We then formulated model selection to choose the right model for the individual in a way that is generalizable and allows for inclusion of additional models if necessary. Similarly, we constructed a model averaging methodology to demonstrate that more accurate predictions can be achieved by averaging models. Our reasons for selecting a *DA framework* were that it allows for incorporation of systems physiology knowledge into the forecast to reduce reliance on dense historical data, and to generate reliable predictions based on sparse data, consistent with existing self-monitoring guidelines and practices, typically available to individuals with diabetes.

We then tested the proposed models using data collected by three individuals with diabetes and two without diabetes. These datasets were selected because they illustrated different self-monitoring scenarios, had different unique properties, and allowed us to test the models under different sets of constraints. In our study, the DA: *(i)* estimated the data well in real time according to standard model evaluation metrics; *(ii)* produced forecasts that compared favorably with the forecasts of certified diabetes educators whose judgment the gold standard for devising personalized nutritional recommendations for individuals with diabetes and represents the best available human-based glucose forecasting; *(iii)* personalized the model to an individual and adapted as the individual changed, providing a personalized medicine approach to diabetes treatment; *(iv)* was integrated with model selection machinery that differentiated model types according to how they estimated the data, allowing a best model to be chosen; *(v)* preformed well given realistic self-monitoring data quality; *(vi)* had accurate output at a high enough frequency, potentially forming the basis for a self-management intervention, and *(vii)* could be averaged in real time to produce even more accurate forecasts.

An ability to accurately forecast postprandial glucose levels has a number of important clinical implications. There exists a considerable body of evidence showing that postprandial hyperglycemia contributes substantially to cardiovascular risks and other complications of diabetes [72], [73]. Previous research argued that an ability to generate accurate predictions of postprandial blood glucose can enable personalized self-management interventions, for example personalized meal planning [31]. We propose that the computational machinery described here could be applied more directly to display glucose forecasts to individuals in order to inform their nutritional choices. Yet communicating predictions with a degree of uncertainty in a useful way is non-trivial, as is evidenced from the extensive research on communicating weather forecasts [74]. In the context of health and health management, the research on communicating risks and uncertainties has primarily focused on informing significant, potentially life-changing decisions, such as surgery, rather than such mundane common decisions as daily meal choices [75]. Moreover, generating glucose forecasts in real time requires accurate macronutrient assessment of meals. Previous research has suggested leveraging crowdsourcing communities for generating timely and accurate nutritional assessments of meals based on these meals’ photographs [76]. Still, more research is needed to identify ways to implement DA-based forecasting to facilitate nutritional decision-making in real-world settings.

Originally designed to guide satellites, control chemical plants and electrical grids, and forecast weather, DA has mostly been applied to *data-rich* situations outside of biomedicine. Inside the biomedical context, DA and control are used in pacemakers, to treat cancer, and to treat patients with type 1 diabetes. The unique advantage of DA is that it infuses data with human knowledge, thus allowing the model to quickly adapt to environmental changes, cope with highly nonlinear systems, and confront forecasting problems, all while *reducing reliance on data in real time*. This is particularly important for such complex multi-dimensional systems as the human endocrine system and blood glucose regulation. Previous research already established feasibility of using machine learning for forecasting individuals’ postprandial blood glucose levels based on nutrition, physical activity, and sleep, while incorporating individuals’ personal clinical and microbiome profiles [31]. Moreover, this work showed that such predictions could be used to inform personalized nutritional interventions. However, because the traditional machine learning approaches do not incorporate knowledge of human physiology, they require massive amounts of relatively clean data that, in the case of diabetes, can only be generated with continuous glucose monitoring. Yet continuous glucose monitoring is not a recommended practice for the vast majority of individuals with diabetes, who continue to rely on infrequent blood glucose measures taken with commercial blood glucose meters. The DA approach is uniquely positioned to reduce the need for data and provide a viable solution with a high translational potential.

The DA methodology can also be used to demonstrate the difficulty of the problem-solving task facing individuals with type 2 diabetes. For the individuals without type 2 diabetes, the point-wise accuracy of the DA forecasts quickly converged to approximately the error present in the glucose measurements—a MSE of 250 translates to about 15 mg/dl of accuracy. In contrast, the point-wise predictive accuracy of the glucose forecasts for the participants with type 2 diabetes was on the order of 500—approximately 22 mg/dl—for both the diabetes educators and the DA. This implies that people with type 2 diabetes seem to face a more difficult glycemic impact prediction task than people who do not have type 2 diabetes, as is reflected in both the diabetes educators and the DA’s ability accurately forecast glucose.

Reaching beyond the direct clinical translation, the DA approach has the potential to forge a deeper understanding of physiology due to its dependency on mechanistic physiology for generating forecasts. The DA allows the ability to exchange and evaluate different mechanistic models with different mechanistic features and observe how adding or subtracting mechanistic components affects forecasts and inferences. In this way the DA enables causal analysis because it allows researchers to *perturb the system* that generates the observable data in time and observe the effects—in other words, perturb the mapping between the mechanistic models that govern the glucose-insulin dynamics [77]. The potential of such causal and comparative analysis is apparent when observing the differences in the forecasts shown in Fig 5 and Table 3. Higher frequency measurements would enable further probing of what physiologic mechanisms are the most important drivers of glucose dynamics. Exchanging different mechanistic engines in the context of DA can promote a better understanding of the mechanisms of the generating process; this goal motivates several data-science challenges. Because forecasts can depend on the DA implementation, there is a need to evaluate the forecasting ability of different DAs. In addition, there is a need for a systematic methodology for relating models with different parameters and mechanisms to one another; if one model with few parameters is a subset of a larger model class, comparing the two only accomplishes a dimensionality reduction of parameter space, and does not probe fundamentally different physiology. Moreover, there is a need for an efficient means of choosing which model parameters to estimate. Finally, there is a need for efficient and accurate methods for patient-specific, real-time optimization of important DA hyper-parameters, such as assumed measurement and process noise.

By design, we restricted our data set to five differently measured, distinct individuals who were all measured relatively sparsely to delve deeply into how DA performs in a practical, patient-level diabetes setting, but this approach has limitations. We could not perform a population-scale analysis, nor could we evaluate which model best reproduced postprandial continuous glucose *dynamics*, as was done with machine learning models in [31]. Nevertheless, we were able to show some boundaries for useful prediction and convergence (about 50 glucose measurements combined with recorded carbohydrates), and we were able to demonstrate how the knowledge embedded in the DA can make up for poor data conditions. Similarly, while the use of a mechanistic model and DA potentially requires substantially less data to achieve an accurate forecast, this choice narrows the ability to fit data and could exclude a good solution if the model cannot represent the physiology of the individual. The fact that forecasts for each individual converged to different levels of error is evidence of these person-specific model errors. Moreover, P3, P4, and P5 converged to mean error rates similar to the error of the glucometers used to make measurements (mean absolute forecast errors were under 15%, which is the maximum error tolerance permitted for most commercially available glucometers).

### Our vision for the use of data assimilation in clinical and biological contexts

#### Alternative methods of parameter estimation

In this paper we chose to use a dual UKF parameter filter to estimate parameters. We made this choice because the UKF has been shown to work well in online situations with minimal tinkering. We could have similarly chosen an Ensemble Kalman Filter [78, 79] or a Particle Filter [5, 79] and those filters would likely work well. It would be useful to compare different filtering techniques, but to do so would require optimizing the filters, a task we purposely did not do because we are focused on applying the filter to a large population soon in a way that can scale large populations. It is likely that there is some space for improvement of parameter estimation using sophisticated methods, such as Markov Chain Monte Carlo methods [55] (MCMC), offline to provide better starting and intermittent parameter estimates.

#### Optimizing parameters, hyperparameters, and properties

The goal of this paper is a proof in principle that we could create an online model-based forecasting machine for predicting glucose for people with type 2 diabetes that is practically scalable to a large population. By doing this we ignored first principles parameter selection, hyperparameter optimization, and general tweaking of the models or DA. This means that there is a lot of space for improvement for all the methods and models. Selecting more useful mechanistic model parameters would improve model and forecasting accuracy. Similarly, adapting noise-related parameters in real time, e.g., *R* and *Q*, or parameters that are sensitive to measurement frequency would likely increase forecast performance. Nearly every UKF hyperparameter could potentially be optimized and many would result in forecast accuracy improvements. The same is true for the non-mechanistic machinery. With the GPMR, for example, it is clear that the training window must be optimized in real-time to make this machine adaptable and useful in real world situations. There is much space remaining for further innovation.

#### Mechanistic model averaging

We demonstrated that it is possible to average models to get a more accurate forecast. We have three comments regarding these results. First, there do exist many different mechanistic glucose-insulin models—for a comprehensive list of linear, nonlinear, and comprehensive models, cf chapter 4 of [8]—therefore it would be relatively easy to add more models to potentially achieve an ever more accurate glucose forecast. Second, we used elementary model averaging machinery. It is easy to imagine using a Bayesian framework for averaging models and their output that would produce improved results, especially when the number of models is increased and may include non-mechanistic models. Third, from the perspective of biological fidelity, it maybe be possible to reduce model error by adding biologically orthogonal models, models that contain weakly interacting mechanisms, or models that operate on different time-scales. We suspect that such an effect is the reason why model averaging worked so well our construction. Specifically, the ultradian model dynamics are on a fast time scale—oscillations on the order of minutes—whereas the meal model tends to capture slower speed dynamics and usually misses the faster oscillatory dynamics. Because of this—in the Fourier-frequency coordinate system sense of breaking the signals down into amplitudes and frequencies of sinusoid functions—these two models are somewhat orthogonal in frequency space. In this way, by averaging in this setting we may be both decreasing model and forecast error; such may not always be the case. Further investigation of this interesting hypothesis will have to wait for another paper.

#### Mechanistic model comparison

It is very difficult to quantify or compare either model’s biological fidelity with the restricted data set we use in this paper—we cannot and do not intend to claim superiority of either model in any objective sense. Similarly, given the limited, clinically realistic data set and complex parameter space where some of the parameters have codependencies, we assume we cannot claim to have achieved a globally optimal model estimate—and such an estimate was not the goal of this paper. The models powering the DA are not general purpose models but mechanistic models with specific physiological constraints. Having more parameters does not translate to complete freedom—in fact, the interrelationships between the parameters of the models are not well understood and can cause obvious problems with parameter estimation. For example, we know that there are parameters we can add to the set being estimated for both models that will, given the data we have, make the MSE forecasts worse and visa versa. Checking all parameter combinations is, especially in real time, hopelessly out of our computational reach. In general, selecting an optimal set of parameters to estimate is a very difficult task—this is why did made our selections based on biological insight constrained by a clinical task. Finally, the models were designed for different purposes, and are, in some sense, orthogonal—we see the shadow of this in the model averaging results where adding the models together made a better forecast implying some degree of independence of the models. We can claim that, relative to a given evaluation metric, particular data set, subset of parameters being estimated, and initial conditions for those parameters, one model can out perform another. This conclusion is supported by Tables 3–5, and even for different metrics, the metrics sometimes conflict as to which model is the most accurate—model selection is evaluation-metric-dependent.

#### Phenotyping and understanding disease progression

A very natural goal using DA and mechanistic modeling is to compute an endocrine-related phenotype defined by the model parameters—doing this and linking it to a genome-wide or phenome-wide association study may help identify diabetes subtypes, help predict onset of type 2 diabetes, and deepen our physiologic understanding of diabetes. While much work has been done in predicting diabetes onset in at-risk adult populations, longer prediction horizons (i.e. identifying latent susceptibilities in young children) would be of exceptional clinical and biological import due to the opportunities for meaningful interventions and the enhanced understanding of long-term endocrine dynamics. Understanding the mechanics of the transition into type 2 diabetes, whether it is simply a smooth change of state or a bifurcation with or without hysteresis, is a different story entirely, but such an understanding requires a strong belief in the biological fidelity of the models. At the heart of this and the phenotyping problem lies the task of addressing model error—or forging an argument that the model and all its parameters do represent the real physiologic processes. To attain adequate biological fidelity, we would likely need to fit all model parameters, control for uncertainty in the parameter estimates, provide external validation, and have mechanistic explanations for the parameter estimates. This task requires more data than is used in this paper because, at the very least, it would require external information—e.g., markers for other physiologic processes such as liver and kidney function—and doing so was neither the goal of, nor within the scope of, this paper.

#### Model error

We have shown that the models personalize to individuals, and there are hints that they do so according to real physiology. We have even seen that Ultradian model error rates for P3, P4, and P5 approach the levels of glucose meter accuracy. Nevertheless, there are obvious limitations to the models implemented—they do not explicitly model many processes known to influence the glucose-insulin system, including stress, exercise, and sleep. Moreover, current models of the glucose-insulin system parameterize nutrition only in terms of carbohydrates, and do not consider the impact of other macronutrients, like protein, fiber, and fat. We have already seen evidence for protein, fiber, and fat-based model errors by identifying significant correlations between forecasting errors and non-carbohydrate meal content. While model improvement is an essential part of accounting for these errors and improving biological fidelity, there also exist opportunites in both machine learning and data assimilation for real-time error correction.

#### Realism and clinical data

Self-monitoring among people with type 2 diabetes is a personal process that has great variability depending on the disease state, personality, and socio-economic status. The data of participants 1 and 2 in this paper represents a realistic best-case well measured patient with type 2 diabetes. A more common data type might be the data of participant 3 who has sparse measurements—sometimes a measurement a day, sometimes a measurement a week, and sometimes several measurements a day. Currently we do not know the bounds of what patients are willing to self-monitor and we do not know the most effective data measurement frequency for powering the DAs. It is clear that machine learning is difficult to use in this context—we could not use participant 3’s data to estimate the GPMR for example because there were not enough data that satisfied the data constraints of the GPMR. We knew about this problem in advance which is why we choose to leverage the physiologic knowledge and online estimation capacity of data assimilation. While it may be tempting to use simulated data to find the optimal measurement frequency or pattern, the problem with using simulated data in this setting is twofold. First, patients do not measure randomly. And second, the real processes driving the physiology are nonstationary and may lie outside what the models typically include—e.g., behaviors such as exercise levels.

#### Filter sensitivity

The results we present in this work are specific to the UKF construction outlined in detail in the appendix. It is important to understand that we can perturb the filtering scheme and get different results; e.g., we know of several filter characteristics we can change that flip which model performs better according to the MSE. We choose the filtering construction not to necessarily optimize the MSE for all people or models, but as a balance between robustness to different people against goodness of fit. But, this brings up two problems that we have attempted to solve by hand but would be valuable problems to solve in a more general setting with a more firm theoretical foundation: robustness of the filter to perturbations of the filter and optimal filtering choice. We do not have good answers for either of these questions from either an analytical or computational framework, but the paper provides clear practical motivation for solving those problems.

#### Nutrition sensitivity

Nutrition is the first order driver of glucose and insulin dynamics. In our study, we established a protocol for nutritionists to follow when estimating the nutritional content of meals and multiple nutritionists estimated meal content for every participant. It is assumed that there is some noise and some bias in the nutritional estimates of the nutritionists, but we do not have a good understanding of the filter sensitivity to nutritionist-generated noise, noise that could be systematic. We did have, in some cases, multiple nutritionists provide nutrition estimates for the same meals and observed a consistency across nutritionists, but if this machinery is made high-throughput, understanding the sensitivity of the filters to nutrition estimates will be important.

### Conclusion

We found that the DA estimated future glucose in a personalized way and showed via model selection that different models have unique advantages, depending on the task (prediction, inference, etc.) and the data source (i.e. patient). There exist certain lower-bounds on data quality for DA-based forecasting, but found these constraints to be mild relative to typical self-management expectations. Overall, we believe that DA-based forecasting is accurate enough, frequently enough to form the basis for a self-management intervention.

## Supporting information

S1 Appendix Dual unscented Kalman filter. (TEX)

S2 Appendix Ultradian endocrine model. (TEX)

S3 Appendix Meal endocrine model. (TEX)
