# Correction: Personalized glucose forecasting for type 2 diabetes using data assimilation

**DOI:** 10.1371/journal.pcbi.1009325

**Published:** 2021-08-20

**Authors:** David J. Albers, Matthew Levine, Bruce Gluckman, Henry Ginsberg, George Hripcsak, Lena Mamykina

S1 Appendix, S2 Appendix, and S3 Appendix appear incorrectly as TEX files. Please view the correct S1–S3 Appendices below as PDF files.

## Supporting information

S1 Appendix Dual unscented Kalman filter. (PDF)

S2 Appendix Ultradian endocrine model. (PDF)

S3 Appendix Meal endocrine model. (PDF)
