# Supplementary material for: Correction: Personalized glucose forecasting for type 2 diabetes using data assimilation
Source: PLoS Comput Biol. 2021 Aug 20;17(8):e1009325. doi: 10.1371/journal.pcbi.1009325 (PMC8378709; doi:10.1371/journal.pcbi.1009325)
Supplement: S1 Appendix — (PDF) [file pcbi.1009325.s001.pdf]

S1 Appendix.

Dual Unscented Kalman Filter

A dual unscented Kalman filter (UKF) was used for simultaneous estimation and tracking of model states and parameters. This methodology approximates the distribution of states by running the model with a simplex of sigma points as initial conditions. The process begins by initializing state and parameter estimates and covariances for a model system with n_x states and n_w estimated parameters.

$$\hat{x}_0 = \mathbb{E}[x_0] \tag{1}$$
$$\hat{w}_0 = \mathbb{E}[w_0] \tag{2}$$
$$P_{x_0} = \mathbb{E}[(x_0 - \hat{x}_0)(x_0 - \hat{x}_0)^T] \tag{3}$$
$$P_{w_0} = \mathbb{E}[(w_0 - \hat{w}_0)(w_0 - \hat{w}_0)^T] \tag{4}$$

Weighting schemes W for determining mean state estimates and covariances were assigned using the methodology described by Wan and van der Merwe [1].
Set weights for the state filter:

$$\lambda_x = \alpha_x^2(n_x + \kappa_x) - n_x \tag{5}$$
$$W_{x,0}^{(m)} = \frac{\lambda_x}{n_x + \lambda_x} \tag{6}$$
$$W_{x,0}^{(c)} = \frac{\lambda_x}{n_x + \lambda_x} + (1 - \alpha_x^2 + \beta_x) \tag{7}$$
$$W_{x,i}^{(m)} = W_{x,i}^{(c)} = \frac{\lambda_x}{2(n_x + \lambda_x)}, \qquad i = 1, \ldots, 2n_x \tag{8}$$

Set augmented weights for the state filter:

$$W_{x,0}^{\text{aug},(m)} = \frac{\lambda_x}{2n_x + \lambda_x} \tag{9}$$
$$W_{x,0}^{\text{aug},(c)} = \frac{\lambda_x}{2n_x + \lambda_x} + (1 - \alpha_x^2 + \beta_x) \tag{10}$$
$$W_{x,i}^{\text{aug},(m)} = W_{x,i}^{\text{aug},(c)} = \frac{\lambda_x}{2(2n_x + \lambda_x)}, \qquad i = 1, \ldots, 4n_x \tag{11}$$

Set weights for the parameter filter:

$$\lambda_w = \alpha_w^2(n_w + \kappa_w) - n_w \tag{12}$$
$$W_{w,0}^{(m)} = \frac{\lambda_w}{n_w + \lambda_w} \tag{13}$$
$$W_{w,0}^{(c)} = \frac{\lambda_w}{n_w + \lambda_w} + (1 - \alpha_w^2 + \beta_w) \tag{14}$$
$$W_{w,i}^{(m)} = W_{w,i}^{(c)} = \frac{\lambda_w}{2(n_w + \lambda_w)}, \qquad i = 1, \ldots, 2n_w \tag{15}$$

UKF State Filter

The state filter of the dual UKF uses the most recent estimates of parameter means w_{k-1}, as well as the most recent estimate of state mean, $\hat{x}_{k-1}$, and state covariance $P_{x_{k-1}}$ to approximate the posterior distribution of the model's mapping of the current state distribution into the future. First, compute sigma points around the current state

estimate:

$$\chi_{0,k-1} = \hat{x}_{k-1} \tag{16}$$
$$\chi_{i,k-1} = \hat{x}_{k-1} + (\sqrt{(n_x+\lambda_x)P_{x_{k-1}}})_i, \qquad i = 1,\dots,n_x \tag{17}$$
$$\chi_{i,k-1} = \hat{x}_{k-1} - (\sqrt{(n_x+\lambda_x)P_{x_{k-1}}})_i, \qquad i = n_x+1,\dots,2n_x, \tag{18}$$

where $(\sqrt{(n_x+\lambda_x)P_{x_{k-1}}})_i$ is the ith column of the matrix square root.
Apply constraints for real positivity to sigma points.

for $j = 1,\dots,n_x$ **do**
  if $\chi^j_{i,k-1} < 0$ **then**
    $\chi^j_{i,k-1} = \min_i \Re(\chi^j_{i,k-1})$
    $\chi^j_{i,k-1} = \max(0, \chi^j_{i,k-1})$
  end if
end for

Compute the posterior sigma points $\chi^*_{i,k|k-1}$ using the mapping (the model) f.

$$\hat{w}_k = \hat{w}_{k-1} \tag{19}$$
$$\chi^*_{i,k|k-1} = f(\chi_{i,k-1}, \hat{w}_{k-1}) \tag{20}$$

Compute the mean and covariance of posterior sigma points $\chi^*_{i,k|k-1}$, and add the assumed model process noise R_v to the posterior covariance estiamte:

$$\hat{x}^*_{k|k-1} = \sum_{i=0}^{2n_x} W^{(m)}_{x,i} \chi^*_{i,k|k-1} \tag{21}$$
$$P_{x_{k|k-1}} = \sum_{i=0}^{2n_x} W^{(c)}_{x,i} (\chi^*_{i,k|k-1} - \hat{x}^*_{k|k-1})(\chi^*_{i,k|k-1} - \hat{x}^*_{k|k-1})^T + R_v \tag{22}$$

Augment posterior sigma points:

$$\chi_{i,k|k-1} = \chi^*_{i,k|k-1}, \qquad i = 0,\dots,2n_x \tag{23}$$
$$\chi_{i,k|k-1} = \chi^*_{0,k|k-1} + (\sqrt{(n_x+\lambda_x)P_{x_{k|k-1}}})_i, \qquad i = 2n_x+1,\dots,3n_x \tag{24}$$
$$\chi_{i,k|k-1} = \chi^*_{0,k|k-1} - (\sqrt{(n_x+\lambda_x)P_{x_{k|k-1}}})_i, \qquad i = 3n_x+1,\dots,4n_x \tag{25}$$

Compute measurement forecast:

$$\hat{x}_{k|k-1} = \sum_{i=0}^{4n_x} W^{\text{aug},(m)}_{x,i} \chi_{i,k|k-1} \tag{26}$$
$$\mathcal{Y}_{k|k-1} = h(\chi_{k|k-1}, \hat{w}_k) \tag{27}$$
$$\hat{y}_{k|k-1} = \sum_{i=0}^{4n_x} W^{\text{aug},(m)}_{x,i} \mathcal{Y}_{i,k|k-1} \tag{28}$$

Compute Kalman gain, where R_n denotes assumed measurement noise.

$$P_{y_k} = \sum_{i=0}^{4n_x} W_{x,i}^{\text{aug},(c)} (\mathcal{Y}_{i,k|k-1} - \hat{y}_{k|k-1})(\mathcal{Y}_{i,k|k-1} - \hat{y}_{k|k-1})^T + R_n \tag{29}$$

$$P_{x_k y_x} = \sum_{i=0}^{4n_x} W_{x,i}^{\text{aug},(c)} (\chi_{\ i,k|k-1} - \hat{x}^*{}_{k|k-1})(\mathcal{Y}_{i,k|k-1} - \hat{y}_{k|k-1})^T \tag{30}$$

$$K_k = P_{x_k y_x} P_{y_k}^{-1} \tag{31}$$

Update state covariance:

$$P_{x_k} = P_{x_{k|k-1}} - K_k P_{y_k} K_k^{-1} \tag{32}$$

Update state estimate:

$$\hat{x}_k = \hat{x}_{k|k-1} + K_k(y_k - \hat{y}_{k|k-1}), \tag{33}$$

where y_k is the kth measurement.

UKF Parameter Filter

The parameter filter of the dual UKF uses the most recent estimates of parameter means, w_{k-1}, and their covariance $P_{w_{k-1}}$, as well as state estimate, $\hat{x}_{k-1}$, to approximate the posterior distribution of parameters, w_k [2]. This is done by creating sigma points that approximate the distribution of w_k, and collecting the measurement forecasts created by the state mappings performed with each of the parameter sigma points, $\mathcal{W}_{i,k|k-1}$.

First, iterate parameter estimates and their covariance:

$$\hat{w}_{k|k-1} = \hat{w}_{k-1} \tag{34}$$

$$P_{w_{k|k-1}} = P_{w_{k-1}} + Q_{w_{k-1}}, \tag{35}$$

where

$$Q_{w_{k-1}} = \text{diag}((\widetilde{\lambda}^{-1} - 1)P_{w_{k-1}}), \tag{36}$$

and diag(·) indicates setting off-diagonal entries to 0.

Compute sigma points around parameter estimate:

$$\mathcal{W}_{0,k|k-1} = \hat{w}_{k|k-1} \tag{37}$$

$$\mathcal{W}_{i,k|k-1} = \hat{w}_{k|k-1} + (\sqrt{(n_w + \lambda_w)P_{w_{k|k-1}}})_i, \qquad i = 1, \ldots, n_w \tag{38}$$

$$\mathcal{W}_{i,k|k-1} = \hat{w}_{k|k-1} - (\sqrt{(n_w + \lambda_w)P_{w_{k|k-1}}})_i, \qquad i = n_w + 1, \ldots, 2n_w \tag{39}$$

Apply constraints for real positivity to sigma points.

for $j = 1, \ldots, n_w$ **do**
  if $\mathcal{W}^j{}_{i,k-1} < 0$ **then**
    $\mathcal{W}^j{}_{i,k-1} = \min_i \Re(\mathcal{W}^j{}_{i,k-1})$
    $\mathcal{W}^j{}_{i,k-1} = \max(0, \mathcal{W}^j{}_{i,k-1})$
  end if
end for

Compute measurement forecast:

$$\mathcal{Y}_{i,k|k-1} = h(f(\hat{x}_{k-1}, \mathcal{W}_{i,k|k-1}), \mathcal{W}_{i,k|k-1}) \tag{40}$$

$$\hat{y}_{k|k-1} = \sum_{i=0}^{2n_w} W_{w,i}^{(m)} \mathcal{Y}_{i,k|k-1} \tag{41}$$

Compute Kalman gain:

$$P_{y_k} = \sum_{i=0}^{2n_w} W_{w,i}^{(c)}(\mathcal{Y}_{i,k|k-1} - \hat{y}_{k|k-1})(\mathcal{Y}_{i,k|k-1} - \hat{y}_{k|k-1})^T + R_n \tag{42}$$

$$P_{w_k y_x} = \sum_{i=0}^{2n_w} W_{w,i}^{(c)}(\mathcal{W}_{i,k|k-1} - \hat{w}_{k|k-1})(\mathcal{Y}_{i,k|k-1} - \hat{y}_{k|k-1})^T \tag{43}$$

$$K_k = P_{w_k y_x} P_{y_k}^{-1} \tag{44}$$

Update parameter covariance:

$$P_{w_k} = P_{w_{k|k-1}} - K_k P_{y_k} K_k^{-1} \tag{45}$$

Update parameter estimate:

$$\hat{w}_k = \hat{w}_{k|k-1} + K_k(y_k - \hat{y}_{k|k-1}), \tag{46}$$

where y_k is the kth measurement.

Table 1. Full list of parameters used in the dual UKF, where the ultradian model has 6 states and the meal model has 12 states. Values for α were determined via ad hoc experimentation, and were important for stable covariance estimates.

**Dual UKF parameters**		
Parameter	Value	Meaning
n_x	12 or 6	Number of model states
n_w	3	Number of estimated model parameters
α_x	0.4	State sigma point weighting constant
α_w	0.3	Parameter sigma point weighting constant
β_x	2	Parameter sigma point weighting constant
β_w	2	State sigma point weighting constant
κ_x	$3 - n_x$	Parameter sigma point weighting constant
κ_w	0	State sigma point weighting constant
$\widetilde{\lambda}$	0.9975	"Forget" factor for parameter covariance
$\mathrm{q_v}$	0.4	State sigma point weighting constant
$\mathrm{q_n}$	0.01	State sigma point weighting constant
R_v	$\mathrm{diag}((\mathrm{q_v} \cdot v_{\mathrm{all}})^2)$	Assumed process noise covariance
R_n	$\mathrm{diag}((\mathrm{q_n} \cdot v_{\mathrm{obs}})^2)$	Assumed measurement noise covariance

The assumed process and measurement noise variance, R_v and R_n, respectively, were computed as fractions ($\mathrm{q_v}$ and $\mathrm{q_n}$) of the time-averaged state values, v_{all}. We considered model process noise to have a standard deviation of 40% of v_{all}. R_n was considered to have a standard deviation of 1% of average measurement values; although typical glucometers have error closer to 10%, we found it advantageous to artificially lower R_n in order to weight measurements more severely and, thus, train the model more quickly. Time-averaged state values, v_{all}, were computed using simulated initial states and nutrition.

Meal Model average state values

$$v_{\text{all}} = \begin{bmatrix} 219.85 \\ 164.08 \\ 8.88 \\ 2.46 \\ 39.26 \\ 37.00 \\ 6307.52 \\ 7914.55 \\ 3103.47 \\ 19.08 \\ 5.413 \\ 0.85 \end{bmatrix} v_{\text{obs}} = 219.85 \tag{47}$$

Ultradian Model average state values

$$v_{\text{all}} = \begin{bmatrix} 82.24 \\ 191.57 \\ 11235.00 \\ 82.02 \\ 82.27 \\ 81.86 \end{bmatrix} v_{\text{obs}} = 11235.00 \tag{48}$$

References

1. Wan EA, Merwe RVD. The Unscented Kalman Filter. In: Kalman Filtering and Neural Networks. Wiley; 2001. p. 221–280.

2. Gove J, Hollinger D. Application of a dual unscented Kalman for simultaneous state and parameter estimation problems of surface-atmospher exchange. J Geophys Res. 2006;111:DO8S07.
