# Supplementary material for: Correction: Personalized glucose forecasting for type 2 diabetes using data assimilation
Source: PLoS Comput Biol. 2021 Aug 20;17(8):e1009325. doi: 10.1371/journal.pcbi.1009325 (PMC8378709; doi:10.1371/journal.pcbi.1009325)
Supplement: S2 Appendix — (PDF) [file pcbi.1009325.s002.pdf]

S2 Appendix.

Ultradian endocrine model

The primary state variables are the glucose concentration G, the plasma insulin concentration I_p, and the interstitial insulin concentration I_i; these three state variables are appended with a three stage filter (h_1, h_2, h_3) which reflects the response of the plasma insulin to glucose levels [1]. The resulting ordinary differential equations take the form [2]:

$$\frac{dI_p}{dt} = f_1(G) - E\left(\frac{I_p}{V_p} - \frac{I_i}{V_i}\right) - \frac{I_p}{t_p} \quad (1a)$$

$$\frac{dI_i}{dt} = E\left(\frac{I_p}{V_p} - \frac{I_i}{V_i}\right) - \frac{I_i}{t_i} \quad (1b)$$

$$\frac{dG}{dt} = f_4(h_3) + I_G(t) - f_2(G) - f_3(I_i)G \quad (1c)$$

$$\frac{dh_1}{dt} = \frac{1}{t_d}\left(I_p - h_1\right) \quad (1d)$$

$$\frac{dh_2}{dt} = \frac{1}{t_d}\left(h_1 - h_2\right) \quad (1e)$$

$$\frac{dh_3}{dt} = \frac{1}{t_d}\left(h_2 - h_3\right) \quad (1f)$$

The *major* parameters include: *(i)* E, a rate constant for exchange of insulin between the plasma and remote compartments; *(ii)* I_G, the exogenous (externally driven) glucose delivery rate; *(iii)* t_p, the time constant for plasma insulin degradation; *(iv)* t_i, the time constant for the remote insulin degradation; *(v)* t_d, the delay time between plasma insulin and glucose production; *(vi)* V_p, the volume of insulin distribution in the plasma; *(vii)* V_i, the volume of the remote insulin compartment; *(viii)* V_g, the volume of the glucose space [3] . $f_1(G)$ represents the rate of insulin production; $f_2(G)$ represents insulin-independent glucose utilization; $f_3(I_i)G$ represents insulin-dependent glucose utilization; $f_4(h_3)$ represents delayed insulin-dependent glucose utilization;

$$f_1(G) = \frac{R_m}{1 + \exp(\frac{-G}{V_g c_1} + a_1)} \quad (2)$$

$$f_2(G) = U_b(1 - \exp(\frac{-G}{C_2 V_g})) \quad (3)$$

$$f_3(I_i) = \frac{1}{C_3 V_g}(U_0 + \frac{U_m - U_0}{1 + (\kappa I_i)^{-\beta}}) \quad (4)$$

$$f_4(h_3) = \frac{R_g}{1 + \exp(\alpha(\frac{h_3}{C_5 V_p} - 1))} \quad (5)$$

$$\kappa = \frac{1}{C_4}(\frac{1}{V_i} - \frac{1}{E t_i}) \quad (6)$$

The nutritional driver of the model $I_G(t)$ is defined over N discrete nutrition events [3], where k is the decay constant and event j occurs at time t_j with carbohydrate quantity m_j

$$I_G(t) = \sum_{j=1}^{N} \frac{m_j k}{60} \exp(k(t_j - t)) \text{where } N = \#\{t_j < t\} \quad (7)$$

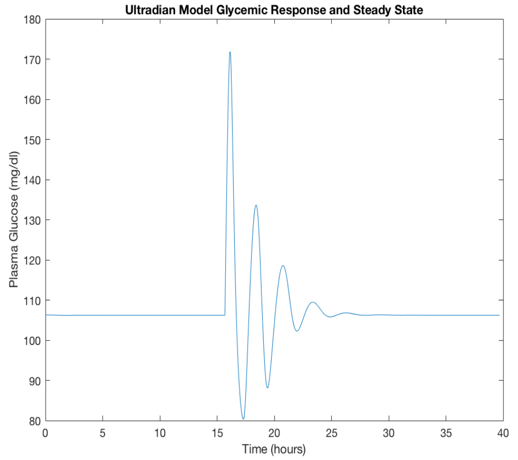

Fig 1. Simulation of post-prandial ultradian glucose oscillations returning to equilibrium. This simulation was performed for a 45 g carbohydrate meal, and employed the initial conditions and parameter values reported by Sturis *et al.* [1]

Table 1. Full list of parameters for the ultradian glucose-insulin model [2]. Note that IIGU and IDGU denote insulin-independent glucose utilization and insulin-dependent glucose utilization, respectively.

**Ultradian model parameters**		
Name	Nominal Value	Meaning
V_p	3 l	plasma volume
V_i	11 l	interstitial volume
V_g	10 l	glucose space
E	0.2 l min^{-1}	exchange rate for insulin between remote and plasma compartments
t_p	6 min	time constant for plasma insulin degradation (via kidney and liver filtering)
t_i	100 min	time constant for remote insulin degradation (via muscle and adipose tissue)
t_d	12 min	delay between plasma insulin and glucose production
k	0.5 min^{-1}	rate of decayed appearance of ingested glucose
R_m	209 mU min^{-1}	linear constant affecting insulin secretion
a_1	6.6	exponential constant affecting insulin secretion
C_1	300 mg l^{-1}	exponential constant affecting insulin secretion
C_2	144 mg l^{-1}	exponential constant affecting IIGU
C_3	100 mg l^{-1}	linear constant affecting IDGU
C_4	80 mU l^{-1}	factor affecting IDG
C_5	26 mU l^{-1}	exponential constant affecting IDGU
U_b	72 mg min^{-1}	linear constant affecting IIGU
U_0	4 mg min^{-1}	linear constant affecting IDGU
U_m	94 mg min^{-1}	linear constant affecting IDGU
R_g	180 mg min^{-1}	linear constant affecting IDGU
α	7.5	exponential constant affecting IDGU
β	1.772	exponent affecting IDGU

References

1. Sturis J, Polonsky KS, Mosekilde E, Cauter EV. Computer model for mechanisms underlying ultradian oscillations of insulin and glucose. Am J Physiol Endocrinol Metab. 1991;260:E801–E809.

2. Keener J, Sneyd J. Mathematical physiology II: Systems physiology. Springer; 2008.

3. Albers D, Elhadad N, Tabak E, Perotte A, Hripcsak G. Dynamical Phenotyping: Using Temporal Analysis of Clinically Collected Physiologic Data to Stratify Populations. PLoS One. 2014;6:e96443.
