# Supplementary material for: Correction: Personalized glucose forecasting for type 2 diabetes using data assimilation
Source: PLoS Comput Biol. 2021 Aug 20;17(8):e1009325. doi: 10.1371/journal.pcbi.1009325 (PMC8378709; doi:10.1371/journal.pcbi.1009325)
Supplement: S3 Appendix — (PDF) [file pcbi.1009325.s003.pdf]

S3 Appendix.

Meal endocrine model

The meal model [1] is composed of two subsystems that represent insulin and glucose kinetics. The glucose subsystem has two compartments, plasma glucose mass G_p and glucose mass in slowly equilibrating tissues G_t. Along with modeling the exchange between these two comparments, subprocesses of the glucose subsystem describe the effects of endogenous glucose production (EGP), rate of glucose appearance (Ra), renal excretion of glucose (E), and insulin-independent and insulin-dependent glucose utilization (U_{ii} and U_{id}, respectively). The insulin subsystem has two main compartments, the insulin masses in plasma and liver (I_p and I_l, respectively). Subprocesses of the insulin subsystem include insulin secretion S and insulin degradation in both the liver and periphery.

Glucose subsystem

$$\begin{cases} \dot{G}_p(t) = EGP(t) + Ra(t) - U_{\mathrm{ii}}(t) - E(t) - k_1 \cdot G_p(t) + k_2 \cdot G_t(t) & G_p(0) = G_{pb} \\ \dot{G}_t(t) = -U_{\mathrm{id}}(t) + k_1 \cdot G_p(t) - k_2 \cdot G_t(t) & G_t(0) = G_{tb} \\ G(t) = \frac{G_p}{V_G} & G(0) = G_b \end{cases} \tag{1}$$

Insulin subsystem

$$\begin{cases} \dot{I}_l(t) = -(m_1 + m_3(t)) \cdot I_l(t) + m_2 I_p(t) + S(t) & I_l(0) = I_{lb} \\ \dot{I}_p(t) = -(m_2 + m_4) \cdot I_p(t) + m_1 \cdot I_l(t) & I_p(0) = I_{pb} \\ I(t) = \frac{I_p}{V_I} & I(0) = I_b \end{cases} \tag{2}$$

Intestinal absorption of glucose ($Ra(t)$)

$$\begin{cases} Q_{\mathrm{sto}}(t) = Q_{\mathrm{sto1}}(t) + Q_{\mathrm{sto2}}(t) & Q_{\mathrm{sto}}(0) = 0 \\ \dot{Q}_{\mathrm{sto1}}(t) = -k_{gri} \cdot Q_{\mathrm{sto1}}(t) + D \cdot d(t) & Q_{\mathrm{sto1}}(0) = 0 \\ \dot{Q}_{\mathrm{sto2}}(t) = -k_{\mathrm{empt}}(Q_{\mathrm{sto}}) \cdot Q_{\mathrm{sto2}}(t) + k_{gri} \cdot Q_{\mathrm{sto1}}(t) & Q_{\mathrm{sto2}}(0) = 0 \\ \dot{Q}_{\mathrm{gut}}(t) = -k_{\mathrm{abs}} \cdot Q_{\mathrm{gut}}(t) + k_{\mathrm{empt}}(Q_{\mathrm{sto}}) \cdot Q_{\mathrm{sto2}}(t) & Q_{\mathrm{gut}}(0) = 0 \\ Ra(t) = \frac{f \cdot k_{\mathrm{abs}} \cdot Q_{\mathrm{gut}}(t)}{\mathrm{BW}} & Ra(0) = 0 \end{cases} \tag{3}$$

Beta Cell Subprocess (insulin secretion is S, and insulin mass in the portal vein is I_{po})

$$S(t) = \gamma \cdot I_{po}(t) \tag{4}$$

$$\dot{I}_{po}(t) = -\gamma \cdot I_{po}(t) + S_{po}(t), \quad I_{po}(0) = I_{pob} \tag{5}$$

$$S_{po}(t) = \begin{cases} Y(t) + K \cdot \dot{G}(t) + S_b, & \dot{G} > 0 \\ Y(t) + S_b, & \dot{G} \leq 0 \end{cases} \tag{6}$$

$$\dot{Y}(t) = \begin{cases} -\alpha \cdot [Y(t) - \beta \cdot (G(t) - h)], & \beta \cdot (G(t) - h) \geq -S_b \\ -\alpha \cdot Y(t) - \alpha \cdot S_b, & \beta \cdot (G(t) - h) < -S_b \end{cases} \tag{7}$$

Renal excretion of glucose

$$E(t) = \begin{cases} k_{e1} \cdot [G_p(t) - k_{e2}], & G_p(t) > k_{e2} \\ 0, & G_p(t) \leq k_{e2} \end{cases} \tag{8}$$

Endogenous glucose production (EGP)

$$EGP(t) = k_{p1} - k_{p2} \cdot G_p(t) - k_{p3} \cdot I_d(t) - k_{p4} \cdot I_{po}(t) \tag{9}$$

$$EGP(0) = EGP_b \tag{10}$$

Hepatic extraction of insulin was fixed, and the following relations were used to evaluate exchange parameters with respect to HE and $S(t)$.

$$\mathrm{HE}(t) = -m_5 \cdot S(t) + m_6, \quad \mathrm{HE}(0) = \mathrm{HE}_b \tag{11}$$

$$m_3(t) = \frac{\mathrm{HE}(t) \cdot m_1}{1 - \mathrm{HE}(t)} \tag{12}$$

Insulin delay

$$\begin{cases} \dot{I}_1(t) = -k_i \cdot [I_1(t) - I(t)] & I_1(0) = I_b \\ \dot{I}_d(t) = -k_i \cdot [I_d(t) - I_1(t)] & I_d(0) = I_b \end{cases} \tag{13}$$

Interstitial Insulin

$$\dot{X}(t) = -p_{2U} \cdot X(t) + p_{2U}[I(t) - I_b] \quad X(0) = 0 \tag{14}$$

Glucose Utilization (Michaelis-Menten kinetics contribute to insulin-dependent glucose utilization)

$$V_m(X(t)) = V_{m0} + V_{mx} \cdot X(t) \tag{15}$$

$$K_m(X(t)) = K_{m0} + K_{mx} \cdot X(t) \tag{16}$$

$$U_{\mathrm{id}}(t) = \frac{V_m(X(t)) \cdot G_t(t)}{K_m(X(t)) + G_t(t)} \tag{17}$$

$$U_{\mathrm{ii}}(t) = F_{\mathrm{cns}} \tag{18}$$

Stomach emptying rate [2]

$$k_{\mathrm{empt}}(Q_{\mathrm{sto}}) = k_{\min} + \frac{k_{\max} - k_{\min}}{2} \cdot \{\tanh[a_{\mathrm{meal}}(Q_{\mathrm{sto}} - b \cdot D)] - \tanh[b_{\mathrm{meal}}(Q_{\mathrm{sto}} - c \cdot D)] + 2\} \tag{19}$$

$$\alpha_{\mathrm{meal}} = \frac{5}{2 \cdot D \cdot (1 - b)} \tag{20}$$

$$\beta_{\mathrm{meal}} = \frac{5}{2 \cdot D \cdot c} \tag{21}$$

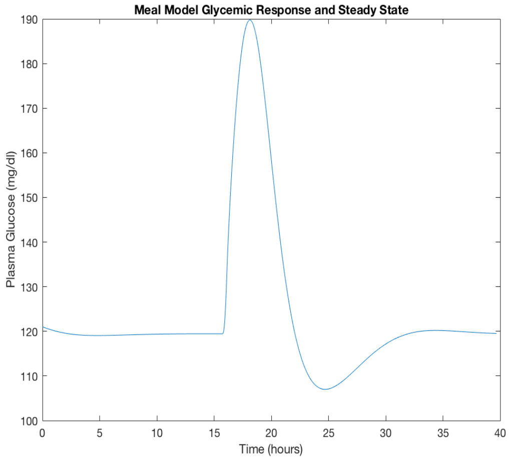

Fig 1. Simulation of post-prandial glucose response and return to equilibrium modeled by the meal model. This simulation was performed for a 45 g carbohydrate meal, and employed the initial conditions and parameter values reported by Dalla Man *et al.* [1]

Table 1. Parameter functions and values for the meal model estimated to best characterize patients with type 2 diabetes by Dalla Man *et al.* .

**Meal model parameters**		
Name	Nominal Value	Meaning
BW	80 kg	Body Weight
V_G	1.49 dl/kg	Plasma glucose space
k_1	0.042 min^{-1}	Glucose exchange rate between rapidly and slowly equilibrating tissues
k_2	0.071 min^{-1}	Glucose exchange rate between rapidly and slowly equilibrating tissues
V_I	0.04 l/kg	Plasma insulin space
m_1	0.379 min^{-1}	Insulin subsystem exchange rate
m_2	0.673 min^{-1}	Insulin subsystem exchange rate
m_4	0.269 min^{-1}	Insulin subsystem exchange rate
m_5	0.0526 min kg/pmol	Insulin subsystem exchange rate
m_6	0.8118	Insulin subsystem exchange rate
HE_b	0.6	Hepatic insulin extraction
k_{max}	0.0465 min^{-1}	Maximum emptying rate of glucose from stomach to intestine
k_{min}	0.0076 min^{-1}	Minimum emptying rate of glucose from stomach to intestine
k_{abs}	0.023 min^{-1}	Intestinal absorption rate
k_{gri}	0.0465 min^{-1}	Rate of Grinding
f	0.90	Fraction of intestinal absorption to appear in plasma
b	0.68	Meal-dose dependency of emptying rate decline
c	0.00023 mg^{-1}	Meal-dose dependency of emptying rate increase
α_{meal}	0.013	Rate of approach to minimum stomach emptying rate
β_{meal}	0.05	Rate of approach to maximum stomach emptying rate

Table 2. Parameter functions and values for the meal model estimated to best characterize patients with type 2 diabetes by Dalla Man *et al.* (continued).

**Meal model parameters cont.**		
Name	Nominal Value	Meaning
k_{p1}	3.09 mg/kg/min	Extrapolated EGP at zero glucose and insulin
k_{p2}	0.0007 min^{-1}	Liver glucose effectiveness
k_{p3}	0.005 mg/kg/min per pmol/l	Amplitude of insulin action on the liver
k_{p4}	0.0786 mg/kg/min per pmol/kg	Amplitude of portal insulin action on liver
k_i	0.0066 min^{-1}	Delay between insulin signal and insulin action
F_{cns}	1 mg/kg/min	Glucose uptake by brain and erythrocytes
V_{m0}	4.65 mg/kg/min	Glucose utilization Michaelis-Menten nominal velocity
V_{mx}	0.034 mg/kg/min per pmol/l	Glucose utilization Michaelis-Menten insulin-dependent velocity
K_{m0}	466.21 mg/kg	Glucose utilization Michaelis-Menten nominal constant
K_{mx}	0 mg/kg	Glucose utilization Michaelis-Menten insulin-dependent constant
p_{2U}	0.0840 min^{-1}	Insulin action rate on peripheral glucose utilization
K	0.99 pmol/kg per mg/dl	Pancreatic responsiveness to glucose rate of change
α	0.013 min^{-1}	Delay between glucose signal and insulin secretion
β	0.05 pmol/kg/min per mg/dl	Pancreatic responsiveness to glucose
γ	0.5 min^{-1}	Exchange rate between portal vein and liver
k_{e1}	0.0007 min^{-1}	Glomerular filtration rate
k_{e2}	269 mg/kg	Renal threshold of glucose

References

1. Man CD, Rizza R, Corbelli C. Meal simulation model of the glucose-insulin system. IEEE Transactions on biomedical engineering. 2007;54:1740–1749.

2. Dalla Man C, Camilleri M, Cobelli C. A System Model of Oral Glucose Absorption: Validation on Gold Standard Data. IEEE Transactions on Biomedical Engineering. 2006;53(12):2472–2478. doi:10.1109/TBME.2006.883792.
